# Revealing Posttranscriptional Regulatory Elements Through Network-Level Conservation

**DOI:** 10.1371/journal.pcbi.0010069

**Published:** 2005-12-09

**Authors:** Chang S Chan, Olivier Elemento, Saeed Tavazoie

**Affiliations:** Department of Molecular Biology and The Lewis-Sigler Institute for Integrative Genomics, Princeton University, Princeton, New Jersey, United States of America; University of Queensland, Australia

## Abstract

We used network-level conservation between pairs of fly *(Drosophila melanogaster/D. pseudoobscura)* and worm *(Caenorhabditis elegans/C. briggsae)* genomes to detect highly conserved mRNA motifs in 3′ untranslated regions. Many of these elements are complementary to the 5′ extremity of known microRNAs (miRNAs), and likely correspond to their target sites. We also identify known targets of RNA-binding proteins, and many novel sites not yet known to be functional. Coherent sets of genes with similar function often bear the same conserved elements, providing new insights into their cellular functions. We also show that target sites for distinct miRNAs are often simultaneously conserved, suggesting combinatorial regulation by multiple miRNAs. A genome-wide search for conserved stem-loops, containing complementary sequences to the novel sites, revealed many new candidate miRNAs that likely target them. We also provide evidence that posttranscriptional networks have undergone extensive rewiring across distant phyla, despite strong conservation of regulatory elements themselves.

## Synopsis

Organisms have evolved extensive regulatory mechanisms for the appropriate expression of genes within precise spatiotemporal contexts. Until recently most of this regulation was thought to be implemented by processes that operate at the “transcriptional” level, that is, by modifying the rate at which mRNA is synthesized. The discovery of short RNAs, termed microRNAs (miRNAs), which can affect gene expression either by degradation of target mRNAs or by inhibiting their translation, has focused much recent effort on determining their specific functional roles and the extent to which they contribute to establishing protein repertoires within individual cells. Chan and colleagues have applied a computational comparative genomic approach for identifying the targets of these miRNAs within 3′ untranslated regions of mRNAs in closely related flies and worms. Their approach identifies a large number of target genes for most of the known miRNAs in these species, providing evidence that these regulators have a much more extensive role than previously thought. The sets of genes targeted by each miRNA are enriched in various known functional classes, providing strong clues for their role in physiology and development. The authors went on to identify many novel miRNAs based on the sequence of highly conserved target sites. They also found a large number of targets that do not correspond to miRNAs, some of which match the targets of known RNA-binding proteins. By comparing the large catalog of putative regulatory elements between flies and worms, they show that, although a large fraction of these elements are conserved, they are targeting, by and large, different sets of genes.

## Introduction

Complex cellular and developmental processes depend on precise spatiotemporal regulation of mRNA and protein levels and activities. Such regulation arises essentially at the transcriptional, posttranscriptional, and posttranslational levels. While tremendous progress has been made in understanding transcriptional regulation and in mapping transcriptional regulatory networks, posttranscriptional regulatory networks are only beginning to be uncovered. Posttranscriptional regulation has been shown to arise through both protein-RNA and RNA-RNA interactions. RNA-binding proteins have been implicated in many aspects of posttranscriptional regulation, e.g., RNA processing, export, localization, degradation, and translational efficiency. Posttranscriptional regulation through RNA-RNA interactions has recently received much attention, in large part due to the discovery of microRNAs (miRNAs) [1].

miRNAs are 21- to 23-nucleotide (nt) single-stranded RNAs, derived from stem-loop precursors. It has been demonstrated that miRNAs regulate mRNA expression either by inducing degradation of the targeted transcript or by decreasing translational efficiency [2]. Recent studies suggest that only the degree of complementarity between a miRNA and its target determines the nature of regulation [2]. Targets with strong complementarity to the miRNA are cleaved by the RNA-induced silencing complex [3]. Such targets appear to be common in plants but rare in animals [4,5]. In some cases, targets with weaker complementarity appear to have decreased translational efficiency, although the molecular mechanism for this repression is currently unknown. It was also recently shown that some miRNAs might be involved in mRNA degradation in animals [6]. Indeed, decreased mRNA levels (in human HeLa cells) were observed for dozens of genes upon transfection of two distinct miRNAs, *miR-1* and *miR-124;* it was also shown that the 3′ untranslated regions (UTRs) of these down-regulated mRNAs have significant complementarity to the 5′ extremity of the transfected miRNAs.

While hundreds of animal miRNAs have been discovered [7], it is likely that many more have not, probably because the conditions under which they are expressed are not known, or because they may be expressed at very low levels. Moreover, very few targets of known miRNAs have been fully characterized experimentally [8]. However, these studies, along with computational ones, indicate that complementarity between miRNAs and their targets is stronger in the 5′ extremity of the miRNAs. Several computational and experimental efforts, based on features of the few verified miRNA/target duplexes, have been directed at finding targets for known miRNAs in the *Drosophila melanogaster* transcriptome [9–11]. In a recent study, human miRNA targets were predicted on the basis of conservation of the seed (the 6-nt sequence at the 5′ extremity of the miRNA) in multiple alignments of five vertebrates [12]. Based on their results, the authors suggest that up to one-third of human genes may be regulated by miRNAs. In another recent study [13], 3′UTR alignments from four mammalian genomes were used to identify highly conserved targets of known miRNAs. Other highly conserved short sequences within their alignments were subsequently used to discover novel miRNAs.

In this paper, we use network-level conservation [14,15] to show that many motifs are highly conserved in the 3′UTRs of orthologous genes from pairs of fly and worm genomes. We show that many of these highly conserved short sequences are complementary to the 5′ extremity of known miRNAs. We show that our approach naturally defines sets of putative target genes for each of these miRNAs, and that some of the target sets are enriched for genes within specific functional categories, shedding new light on miRNA involvement in these processes. Our approach also discovers known sites for RNA-binding proteins, motifs known to be involved in mRNA decay in other species, and many novel sites that are strongly associated with specific functional enrichments. We show that some of the highly conserved sites are often simultaneously conserved within the same 3′UTRs, suggesting combinatorial regulation of these transcripts. Since our approach uncovers many sites that are not known to be targeted by miRNAs or RNA-binding proteins, we describe a simple approach for discovering new miRNAs in the worm and fly genomes, and show that the candidate novel miRNAs have all the features of known miRNAs.

## Results

### Scoring Exhaustive Motif Lists for Network-Level Conservation

**Figure 1:**
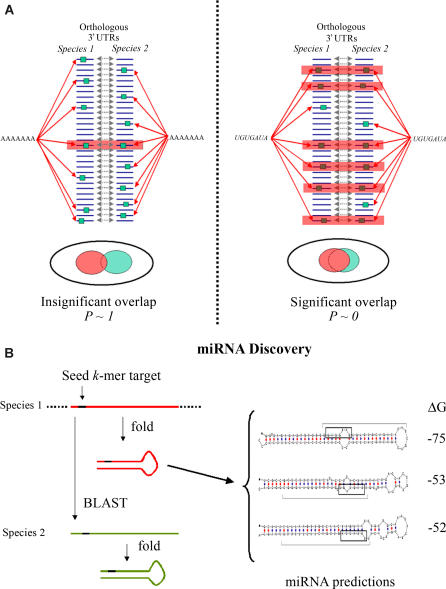
Schematic Representation of the Approach (A) In the first stage of our approach, we scored exhaustive lists of *k*-mers for network level conservation. Schematic examples for a nonconserved *k*-mer (AAAAAAA) and a highly conserved one (UGUGAUA) are given in the left and right graphics, respectively. (B) In the miRNA discovery stage, seed *k*-mers are used to search the genome for conserved and stable stem-loops.

We modified FastCompare [15], for processing mRNA sequences (i.e., we performed single-strand analyses), to calculate a conservation score for all 7-, 8-, and 9-mers from the 3′UTRs of worm and fly genes. Briefly, a *k*-mer is given a high conservation score if there is a significant overlap between the sets of orthologous genes having at least one copy of the *k*-mer anywhere in their 3′UTRs. The hypergeometric distribution is used to evaluate the significance of the overlap. Conservation scores are defined as the negative logarithm of the cumulative hypergeometric *p*-values (see Figure 1A and Materials and Methods). However, the hypergeometric *p*-values are only treated as relative measures of conservation, and are not used in the traditional null hypothesis rejection scheme. Further details can be found in Materials and Methods and in [15].

**Figure 2:**
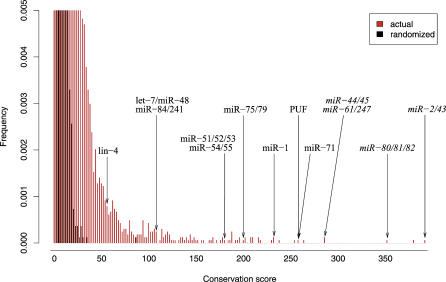
Distribution of Conservation Scores for the *C. elegans*/*C. briggsae* Analysis on 3′UTR Sequences Distributions of actual (red) and randomized (black) sequences are shown. Scores corresponding to some of the known miRNA target sites and RNA-binding protein sites in worms are indicated by arrows. The top portion of both distributions are not shown, for the purpose of presentation.

As a control, we applied FastCompare to sets of randomized 3′UTRs with the same length and same level of divergence as the original sequences [15]. Figure 2 shows the distribution of conservation scores for all 7-mers in worms, obtained for real data and a single randomized control; it clearly shows that the extremely high conservation scores obtained on real data are very unlikely to be obtained by chance. The same pattern was observed for flies (Figure S1). We retained the top 500 7-mers (3.5% of all 16,384 7-mers) for further analysis (see below for a justification of this cutoff, in terms of number of captured miRNAs). We determined that the conservation score threshold induced by retaining the top 500 7-mers is, on average, greater than 99.9% of the scores obtained from randomized data. Using the procedure described in Materials and Methods, we mapped the 500 7-mers into 442 *k*-mers for worms and 497 for flies (with *k* = 7, 8, or 9). We observed that, in both cases, the list of highest-scoring *k*-mers often contains several overlapping, slightly distinct variants of the same sites, as shown for some of the highest-scoring worm *k*-mers in Table S1.

We provide two lines of evidence that the high-scoring *k*-mers obtained in this study are not DNA regulatory elements (transcription factor binding sites [TFBSs]). First we determined that the overlap between the highest-scoring *k*-mers obtained in the present study and the ~400 highest-scoring *k*-mers found in the analysis of 2-kb upstream regions in the same worm and fly genomes [15] is very small (15 for worms, seven for flies). Since TFBSs are most abundant in upstream regions (see [16] for a review), this provides supportive evidence for the limited presence of TFBSs among our highest-scoring *k*-mers; although we cannot rule out the presence of unique transcription factor binding sites that function exclusively within 3′ downstream regions. Second, we reasoned that, if our highest-scoring *k*-mers were TFBSs, they would generally not have any strand bias, i.e., a *k*-mer and its reverse complement should be roughly equally conserved. However, we found that 97% (worm) and 90% (fly) of our highest-scoring *k*-mers have a higher conservation score than their reverse complement (which are themselves generally not among our highest-scoring *k*-mers). As an example of highly significant strand bias, the highest-scoring worm *k*-mer, CUGUGAU, is conserved in 187 genes, while its reverse complement, AUGACAG, is conserved in only 16 genes.

### High-Scoring *k*-Mers Are Complementary to the 5′ Ends of Many miRNAs

We observed that the highest-scoring fly *k*-mer, UGUGAUA, corresponds to the K box, a short sequence that has been found in the 3′UTRs of many genes of the *E(spl)* complex in *D. melanogaster* [17]; this sequence has been shown to reduce mRNA transcript levels in vivo, and to a lesser extent, to also reduce protein levels [17]. Another short sequence with a verified posttranscriptional role [18,19], the Bearded (Brd) box (AGCUUUA, rank 23) was also identified as a highly conserved motif. These two motifs were shown to be complementary to the 5′ extremity of several fly miRNAs [20]. We then systematically matched our highest-scoring worm and fly *k*-mers to the 117 *Caenorhabditis elegans* and 79 *D. melanogaster* known (and experimentally verified) miRNAs, from the miRNA registry [7].

**Figure 3:**
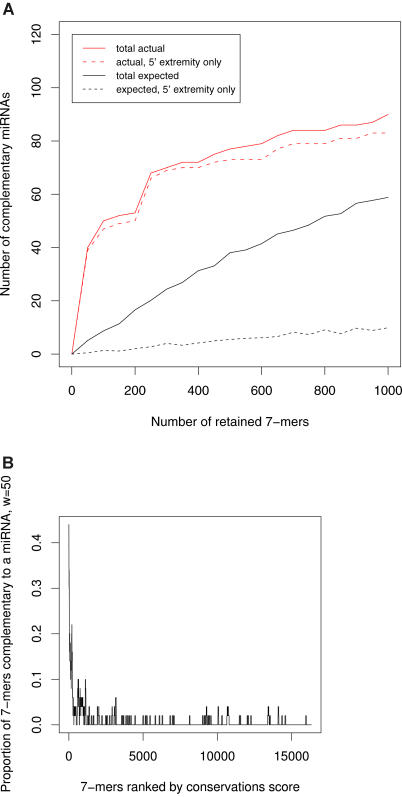
High-Scoring *k*-Mers Are Complementary to the 5′ Ends of Many miRNAs (A) Number of complementary worm miRNAs as a function of initial number of retained 7-mers. Solid lines correspond to complementarity anywhere within the miRNAs. Dashed lines correspond to complementarity to the 5′ extremity of miRNAs only. Complementarity to the 5′ extremity of a miRNA is defined as starting within 1 nt of the actual miRNA 5′ extremity. (B) Proportion of 7-mers complementary to the 5′ extremity of at least one miRNA, as a function of the conservation rank (using a sliding window [w] of size 50).

**Figure 4:**
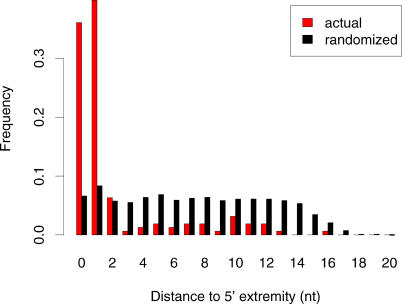
Distribution of Distances from the First Nucleotide of the *k*-Mer to the 5′ Extremity of the miRNA Distances are given for all pairs of high-scoring *k*-mers/complementary miRNAs. The distribution clearly shows that complementarity between high-scoring worm *k*-mers and miRNAs occurs primarily at the 5′ extremity of the miRNAs.

We found that 87 and 73 of our 442 and 497 highest-scoring worm and fly *k*-mers (respectively) had perfect complementarity to at least one known miRNA, and that, conversely, 77 and 57 different miRNAs were complementary to at least one high-scoring *k*-mer. The expected numbers of miRNAs matched by chance are significantly lower (approximately 38 and 24 for worm and fly, respectively; see Figures 3A for worms and S2A for flies; see Materials and Methods for explanations). However, we found that the vast majority of *k*-mers matched miRNAs within their 5′ extremity (see Figures 3A and 4 for worms, and Figures S2A and S3 for flies). Note that here, and in the rest of this study, we define complementarity to the 5′ extremity of a miRNA as complementarity starting within 1 nt or less of the actual miRNA 5′ extremity (e.g., positions 1 or 2 of the miRNA). Of the *k*-mer/miRNA pairings, 76% and 67% occur within the miRNA 5′ extremity, and the number of distinct miRNAs that are complementary to at least one *k*-mer within their 5′ extremities is 73 for worms and 49 for flies; this represents 62.4% and 62.0% of all known and experimentally verified miRNAs in *C. elegans* and *D. melanogaster,* respectively. For both worms and flies, the expected number of miRNAs whose 5′ extremity is complementary to the same number of *k*-mers selected at random is small: 5 for worms and 3.5 for flies (see Figures 3A and S2A), signifying that only a small proportion (6.8% and 7.1%) of the captured miRNAs is expected to be due to chance. Figure 3A shows that significantly increasing the initial number of retained worm 7-mers (we currently retain the 3.5% highest-scoring 7-mers) would yield very few additional complementary miRNAs; however, it would significantly increase the number of complementary miRNAs expected by chance. The same holds for flies (see Figure S2A).

Interestingly, almost all the *k*-mers that are complementary to miRNAs are 7-mers (i.e., they were not extended into 8-mers). We observed that, for most worm miRNAs in this study (58/73), 7-mers that are complementary to positions 2–8 of miRNAs are more conserved than 7-mers complementary to positions 1–7. Intriguingly, the situation was almost opposite in flies, with only 18/49 miRNAs having a more conserved complementary 7-mer in positions 2–8.

We also investigated whether highly conserved *k*-mers that are not exactly complementary to the 5′ extremity of any miRNA can still pair with certain miRNAs, if we tolerate a single non-Watson-Crick GU pairing. We found that 41 highly conserved *k*-mers, complementary to 53 distinct miRNAs, fit that scenario in worms. This number of *k*-mers is much larger than the average (approximately 11) obtained when we start from the same number of randomly selected *k*-mers (repeated 100 times). Interestingly, out of these 53 complementary miRNAs, 45 (85%) are also exactly complementary (in their 5′ extremity) to one of our high-scoring *k*-mers. This suggests that at least certain miRNAs in worms can bind their targets either through exact complementarity or through inexact complementarity involving a small number of GU pairs. The high network-level conservation of certain *k*-mers with imperfect complementary to miRNAs may indicate that miRNA targets involving imperfect pairing through GU pairing constitute a functionally distinct class of targets, similar to observations made for transcription factors in bacteria [21]. The same analysis in flies yielded 17 *k*-mers, complementary (through one GU pairing) to 19 miRNAs. This number of *k*-mers was closer to the expected number (approximately nine) obtained from randomly selected *k*-mers, than in the worm analysis. This suggests that targets involving GU pairing may be less common in fly than in worms.

Since the signal-to-noise ratio appears to be much higher when considering only exact complementarity to miRNAs (in worms, 57 *k*-mers are exactly complementary to the 5′ extremity of known miRNAs, with only 4.3 expected by chance), we restricted the rest of our analyses to such exact complementarity. Nonetheless, the list of *k*-mers/complementary miRNAs with one GU pairing is available from our Web site (http://tavazoielab.princeton.edu/mirnas/).

In Figure 3B we show the proportion of 7-mers (within a sliding window of 50 7-mers) that are (exactly) complementary to the 5′ extremity of at least one miRNA as a function of the conservation score rank in worms. As can be seen in the figure, complementarity to the 5′ extremity of a miRNA correlates very strongly with conservation at the network level. A similar correlation was observed for flies (see Figure S2B).

The known *C. elegans* miRNAs with 5′ complementarity to at least one *k*-mer are shown in Table 1. The same information for flies is shown in Table S2. It is interesting to note that many of the highest-scoring worm *k*-mers that are complementary to known miRNAs are also highly conserved in flies, and vice versa. Moreover, worm *k*-mers that are highly conserved in flies are almost always complementary to the 5′ extremity of at least one fly miRNA (see Table 1). Table S3 shows the few miRNAs with complementarity to a highly conserved *k*-mer not occurring at the 5′ extremity. These cases may be due to chance; alternatively, they may be due to slightly erroneous annotation of the mature miRNA boundaries, or they may signify that some miRNAs are not restricted to recognizing their targets through their 5′ extremity.

**Table 1:**
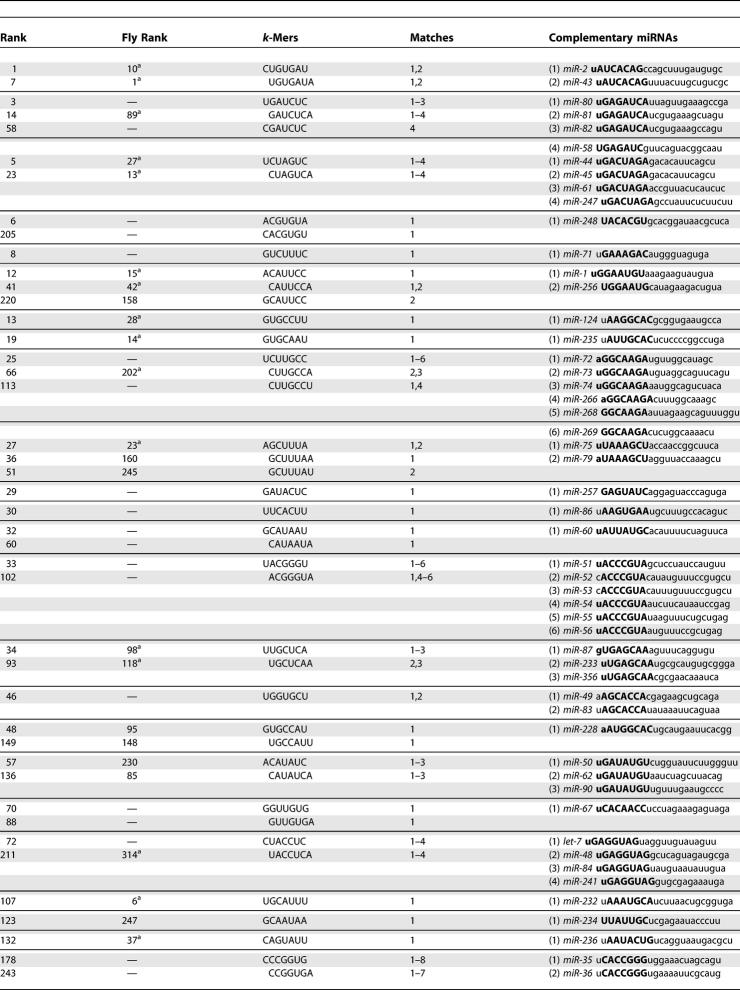
Worm *k*-Mers Complementary to 5′ Extremity of Known Worm miRNAs for 73 Distinct miRNAs

**Table 1:**
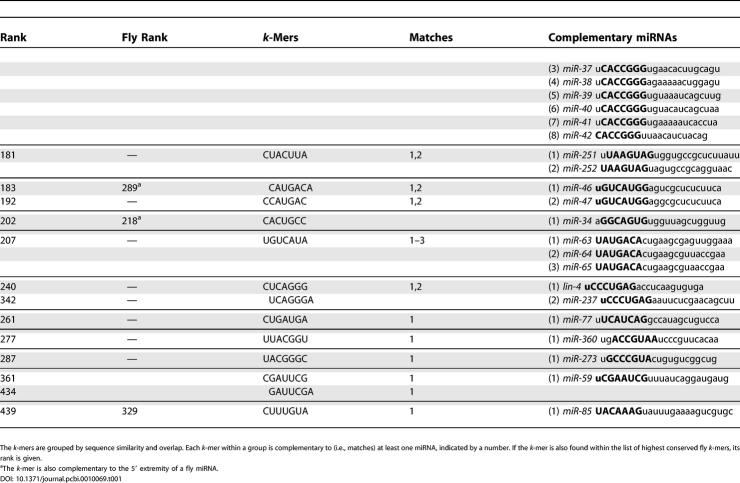
Continued

### Prediction and Analysis of miRNA Targets

The observations above suggest that the presence of a conserved *k*-mer within the 3′UTR of a given gene indicates targeting and regulation by the miRNA whose 5′ extremity is complementary to the *k*-mer. Our approach thus conveniently defines sets of putative targets for each miRNA: A gene is predicted to be a target of a given miRNA if its 3′UTR and that of its ortholog contain a high-scoring *k*-mer that is complementary to the 5′ extremity of the miRNA. Note that a similar approach for defining miRNA targets has been described [12,13,22,23], in which targets were defined as short sequences conserved within multiple alignments of several 3′UTR sequences from closely related species (vertebrates and flies). For flies, we observed that many of the largest 3′UTRs correspond to genes involved in development (for example, the 200 genes with largest 3′UTRs are strongly associated with the organ development Gene Ontology [GO] category, *p* < 10^−19^). To avoid systematically biasing our predicted targets toward these genes, we used real-length 3′UTRs when the length is less than 500 nt, but truncate larger 3′UTRs to 500 nt. Although many real targets are likely to be located beyond the 500-nt cutoff, we expect most of them to be retained (80% of annotated fly 3′UTRs are less than 500 nt). For worms, we used the real-length 3′UTRs. Although few miRNA targets have been experimentally verified, our predicted targets include some for which experimental evidence is available. For example, the predicted targets for worm *let-7* includes *hbl-1,* a gene that is likely regulated by *let-7* [24]. As another example, recent in vitro and in vivo experiments suggest that members of the fly *miR-2* family *(miR-2a/2b/2c)* regulate the proapoptotic genes *reaper, grim,* and *sickle* in *D. melanogaster* [9]. Indeed, our predicted target set for *miR-2a/2b/2c* (259 genes having a conserved CUGUGAU or UGUGAUA in their 3′UTRs) contains the *reaper* and *sickle* genes (but not *grim*), as well as several other genes known to be involved in apoptosis: *CG10345, CG11593, tartan, croquemort,* and *Ice*. These genes are not yet known to be miRNA targets, and therefore constitute strong candidates for experimental verification of regulation by members of the *miR-2* family.

We found that many miRNAs are also associated with significant functional enrichment(s) (see Table 2 for worms and flies). For example, we found that the predicted target set of *C. elegans miR-1* (197 genes having a conserved ACAUUCC or CAUUCCA in their 3′UTRs) contains many genes involved in proton transport (*p* < 10^−10^) and ATPase activity (*p* < 10^−9^). In fact, most of the mRNAs encoding cytosolic sector subunits (A, B, D, E, F, and H) of a *C. elegans* vacuolar H^+^-ATPase contain a conserved target site for *miR-1,* suggesting a miRNA-mediated regulation of this proton-pumping complex.

**Table 2:**
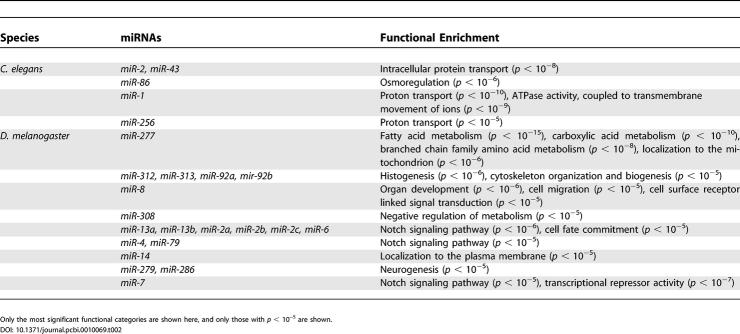
Functional Enrichments for Some of the Known *C. elegans* and *D. melanogaster* miRNA Target Sets

In flies, the predicted target set for *miR-2a/b/c* is enriched with genes annotated in GO as involved in the Notch signaling pathway (*p* < 10^−6^). In previous studies, the K box was found in the 3′UTR of many members of the *E(spl)* and *Brd* gene complexes, which are targets of the Notch signaling pathway [17,20,25]. Indeed, the target set for *miR-2a/b/c* contains the *Brd* genes *m2, m4,* and *mα* and the *E(spl)* genes *m3, m5, mδ,* and *E(spl).* It also contains the *fringe* and *serrano* genes, which are other known components of the Notch pathway (note that these two genes were also predicted as targets in [9,10]). The target sets for the miRNAs targeting the Brd box, *miR-4* and *miR-79*, are also enriched with genes involved in the Notch signaling pathway (*p* < 10^−5^ in both cases).

The predicted target set for worm *miR-277* (the union of conserved sets for GCAUUUA, UGCAUUU) is highly enriched with fatty acid metabolism (*p* < 10^−15^), carboxylic acid metabolism (*p* < 10^−10^), and branched chain family amino acid metabolism (*p* < 10^−8^). In a recent computational study, several enzymes of the branched chain amino acid degradation pathway were proposed to be targets for *miR-277* [9]. The functional enrichment of its target set suggests a much broader role for *miR-277,* perhaps acting as a general metabolic switch, slowing down metabolic activity by repressing translation of these genes.

**Figure 5:**
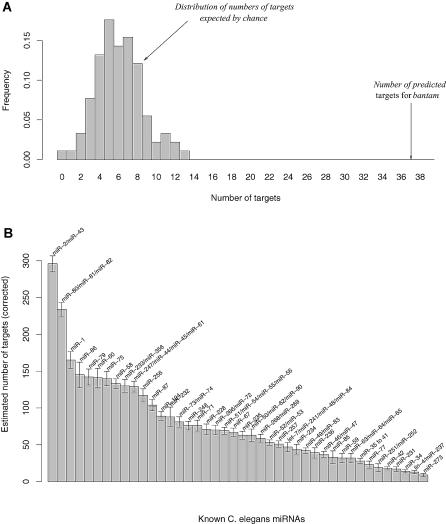
Number of miRNA Targets (A) Example showing that the number of predicted targets for *D. melanogaster bantam* is much larger than expected by chance. The number of predicted targets is the number of genes whose 3′UTR contains at least one conserved *k*-mer complementary to the 5′ extremity of the corresponding miRNA. The distribution of numbers of targets expected by chance was obtained by running the same analysis using 100 pairs of randomized genomes with the same level of divergence as the original ones (see Materials and Methods for details). (B) Estimated numbers of targets for *C. elegans* miRNAs (only for miRNAs that are complementary to at least one of our high-scoring *k*-mers). Each number corresponds to the number of predicted targets (as defined above) minus the average number of targets expected by chance over the 100 randomizations. The error bars correspond to two standard deviations.

We used conserved sets obtained from randomized sequences to show that the number of targets we predict is much larger than the number expected by chance (see Figure 5A for an example with *bantam,* a *D. melanogaster* miRNA). The expected number of targets provided us with an estimate of the number of false positives in our sets of targets. Once sets of targets have been corrected for false positives, our approach could then provide insights into the topology of miRNA regulatory networks in metazoan genomes. For example, Figure 5B shows that some worm miRNAs potentially regulate hundreds of genes (e.g., *miR-2*/*miR-43*), while others may regulate fewer than ten genes (e.g., *miR-273*). Most miRNAs appear to regulate between 50 and 100 genes both in worms and flies (see Figures 5B and S4), a number that agrees with other recent estimates [8].

To further validate our sets of predicted targets, we investigated whether coexpressed genes are regulated by the same miRNAs. When using the *C. elegans* early embryonic microarray time-course [26], we found that 3,131 pairs of highly coexpressed genes (Pearson correlation ≥ 0.8) contain at least one predicted target for the same miRNA. Using randomizations, we calculated that this number is significantly higher than expected by chance (*p* < 0.044), thus providing statistical evidence that the same miRNAs tend to regulate mRNAs that are coexpressed (at least during *C. elegans* early embryogenesis).

Finally, we generated the subsets of target genes for which high-scoring *k*-mers are also conserved within global alignments of the 3′UTRs (we used CLUSTALW with default parameters to generate the alignments). The list of these target genes is available on our Web site (http://tavazoielab.princeton.edu/mirnas/). On average, 49% and 75% of our initial target predictions correspond to *k*-mers at the same position in these alignments, in worms and flies, respectively. These subsets thus contain predicted target sites that are further constrained. Although nonaligned predicted targets may contain many false positives, we suspect that small scale DNA rearrangements, fast evolution of noncoding sequences, or imprecise definition of 3′UTR boundaries may, in many cases, make alignment-based methods unreliable. Our global list of predicted targets may therefore contain many functional targets that will not be found using traditional alignment-based approaches.

### Biological Significance of Other High-Scoring *k-*Mers

Many of our high-scoring *k*-mers are not complementary to any known miRNA. For example, we found several AU-rich motifs that are highly conserved both in worms and flies, e.g., UAAUUUAU (ranks 4 and 11 in worms and flies, respectively) and UAUUUAUU (ranks 6 and 2). These motifs are similar to the AU-rich element (ARE), defined as UUAUUUAUU [27]; the ARE was found in the 3′UTRs of cytokines and proto-oncogenes in human [28]. It was shown to destabilize these mRNAs at least in part by triggering rapid deadenylation [27]. AREs have not been shown to be functional in worms or flies; however, a chimeric mRNA consisting of the rabbit β-globin gene fused to the 3′UTR of the human TNF-α (which contains several AREs) was rapidly degraded in *Drosophila* S2 cells [29]. Moreover, this degradation involves homologs of human genes known to be involved in ARE-mediated mRNA decay [29]. Interestingly, it was shown in the same study that the human *miR-16* miRNA is required for ARE-mediated mRNA turnover. In worms, the genes whose 3′UTR contains conserved UAAUUUAU appear to be enriched for genes whose products localize to the endoplasmic reticulum (*p* < 10^−11^) and to the proteasome complex (*p* < 10^−10^); for example, eight (out of 14) genes encoding products that localize to the core proteasome complex contain a conserved UAAUUUAU in their 3′UTRs.

We also found that UGUAAAUA, a sequence bound by some members of the PUF family, was highly conserved both in worms and flies (ranks 9 and 4, respectively). Interestingly, as we will see below, the PUF binding site appears to be better represented by a gapped motif in worms. *D. melanogaster* possesses a single PUF protein named Pumilio. Early in embryogenesis, Pumilio controls anterior/posterior body patterning by binding to the 3′UTR of *hunchback* mRNA and repressing its translation (via interaction with Nanos) [30]. There is strong evidence that Pumilio also inhibits pole-cell division in early embryogenesis by repressing the translation of *cyclin B* [31]. Finally, Pumilio has also been shown to be involved in neuronal excitability [32], long-term memory [33], and dendrite neurogenesis [34]. However, no additional targets have been identified yet. Altogether, these studies suggest that Pumilio targets many mRNAs, with potentially a small fraction of them having been identified. The fly conserved set for UGUAAAUA contains 314 genes, thus providing a large number of potential targets awaiting experimental verification; it would be particularly interesting to focus experiments on genes that are expressed early in embryogenesis (maternal and zygotic) and genes expressed in the brain.

Finally, we found many highly conserved *k*-mers that are not yet known to be bound by any RNA-binding proteins (and are not complementary to any known miRNAs), but which are associated with strong functional enrichment. In worms, the sequences UUGUUGA, UGUUGUU, and UUGUUAU appear to be highly conserved in the 3′UTRs of many genes involved in cell growth (*p* < 10^−17^, *p* < 10^−23^, and *p* < 10^−24^, respectively). Indeed, out of the 497 genes containing a 3′UTR with a conserved UUGUUGA, UGUUGUU, or UUGUUAU, 192 are annotated as being involved in growth (*p* < 10^−49^, 64 expected). Moreover, the protein products of 60 of these 497 genes are known to localize to the ribosome (*p* < 10^−49^, six expected by chance). The same elements are also conserved downstream of many genes involved in larval development (*p* < 10^−38^) and gametogenesis (*p* < 10^−15^). This motif may be involved in slowing down cell growth by repressing translation or degrading large numbers of mRNAs at a certain developmental stage, or under stressful environmental conditions.

In flies, CAG-repeats (CAGCAGC, rank 71; and GCAGCAG, rank 104) are strongly associated with genes involved in transcriptional regulation (*p* < 10^−14^ for both *k*-mers). For example, among the 14 genes that have a conserved motif consisting of four tandem copies of CAG, six are known transcription factors *(fork head, ventral veins lacking, SoxNeuro, cropped, spineless,* and *ypsilon schachtel),* and two are transcriptional co-activators or co-repressors *(big brother, smrter)*. CA repeats are also highly enriched with genes involved in organ development (*p* < 10^−11^ for CACACAC, rank 32). There is growing evidence that CA repeats are involved in mRNA transcript stability, at least in human. For example, in one recent study it was shown that CA repeats in the 3′UTR of the *bcl-2* mRNA are responsible for destabilization of the transcript [35]. In another study, CA repeats within intron 13 of the human endothelial nitric oxide synthase gene were found to mediate cleavage of the pre-mRNA, unless bound by heterogeneous nuclear ribonucleoprotein L [36]. Although experiments are needed to validate our observations, these results provide strong support for the functionality of certain classes of repeats in posttranscriptional regulation.

### Motifs Represented by Gapped *k*-Mers Often Show Stronger Conservation than Ungapped *k*-Mers

The binding sites for the PUF RNA-binding proteins in yeast are **UGUA..UA**, **UGUA...UA**, and **UGUA....UA** for Puf3p, Puf4p, and Puf5p, respectively [37]. This means that UGUA and UA are required for binding to the PUF proteins, but also that the nucleotides between the two half-sites are less important (although nucleotide preferences still exist within the gaps [37]. In yeast, the length of the gap between UGUA and UA determines which PUF protein binds the site with highest affinity. In what follows, we refer to such elements as “gapped” *k-*mers. To search for these regulatory elements within the 3′UTRs of worm and fly mRNAs, we calculated a conservation score for all sequences of the form *s_1_*-*gap*-*s_2_,* where the lengths of *s_1_, gap,* and *s_2_* vary between 2 and 4 nt. We found that 157 (worm) and 215 (fly) gapped *k*-mers were more conserved (in terms of network-level conservation score) than any of the ungapped *k*-mers they matched. Although some of these gapped *k*-mers are complementary to some miRNAs, the position at which complementarity begins is less biased than for ungapped *k*-mers: Out of 52 gapped *k*-mer/miRNA pairs in worms, only 17 (33%) occur within 1 nt or less from the 5′ extremity of the miRNAs. The same holds for fly, in which out of 26 gapped *k*-mer/miRNA pairs, only ten (38%) occur within 1 nt or less from the 5′ extremity of the miRNAs. Some of the highest-scoring worm gapped *k*-mers are shown in Table 3.

**Table 3:**
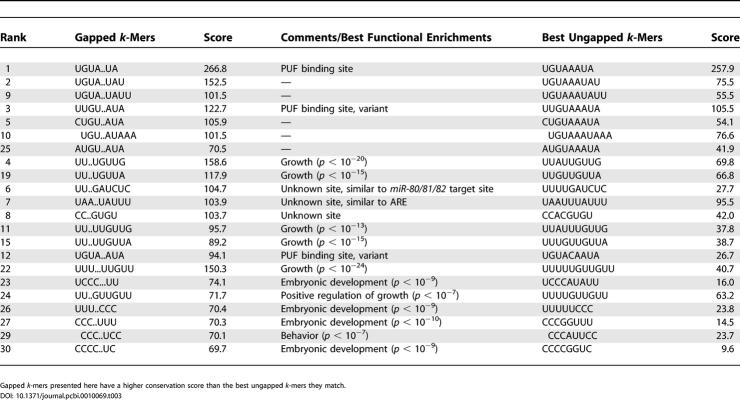
Selection of Highest-Scoring Gapped *k*-Mers in Worms

The highest-scoring gapped *k*-mer in worms, **UGUA..UA**, matches the experimentally defined binding sites for Puf3p in yeast [37]) and mouse PUM-2 (consensus **UGUA.AUA** [38]). Although in yeast, Puf3p targets the mRNA of many genes encoding proteins that are localized to the mitochondrion, in worms we do not find any particular functional enrichment using the GO annotations. The *C. elegans* genome contains eight distinct PUF proteins, although some of them duplicated recently and might be redundant [39]. FBF-1 and FBF-2 (93% identical) regulate the germ line switch from spermatogenesis to oogenesis by posttranscriptionally repressing *fem-3* [40]*.* Although the nature of this repression is unknown, the required physical interaction between FBF and NANOS-3, a homolog of *Drosophila* Nanos, suggests that FBF represses the translation of *fem-3* [41], as Pumilio does for *hunchback.* Table 3 contains several motifs that also resemble the PUF binding site (e.g., **UUGU..AUA** and **UGUA..AUA**); these motifs could be bound by other members of the PUF family in *C. elegans*.

Several of the highest-scoring gapped *k*-mers in worms are variants of the growth-related motifs described above (e.g., **UU..UGUUG**, **UU..UGUUA**; see Table 3) and have similar functional enrichments. Several other highly conserved gapped *k*-mers appear to be involved in embryonic development, e.g., **UUU..CCC** and **CCC..UUU** (*p* < 10^−9^ and *p* < 10^−10^, respectively).

### Coregulation by Multiple Target Sites

Simultaneous conservation of two distinct high-scoring *k*-mers (termed conserved co-occurrence) provides a simple way to discover putative coregulation by pairs of regulatory elements (or, more precisely, between the molecules that bind them). Pair members of high-scoring *k*-mers that differ in at least 3 nt were scored and sorted according to network-level conservation, as described above for single elements. Only pairs of *k*-mers conserved in at least ten genes were retained for further analysis. For the most conserved co-occurrences, we also calculated the statistical significance of the overlap between the conserved sets corresponding to each element taken separately (unlike for network-level conservation, we assumed that these conserved sets were approximately independent, provided that the *k*-mers were different enough in sequence). In both worms (Table 4) and flies (Table S4), we found that many distinct miRNA target sites are strongly co-conserved. For example, in worms, the target sites for *miR-75/79* and *miR-86* are simultaneously conserved in the 3′UTRs of 16 genes (*p* < 10^−9^). Similarly, the target sites for *miR-2/43* and *miR-1* are coconserved in 12 genes (*p* < 10^−6^). Consistent with previous observations [17], target sites for *miR-2a/2b/2c/6/13a/13b* (UGUGAUA, K box) and *miR-277* (AGCUUUA, Brd box) are significantly coconserved within fly 3′UTRs (*p* < 10^−17^). As shown in Tables 4 and S4 for both worms and flies, miRNA target sites are very often coconserved with AU-rich elements, potentially linking miRNA-based regulation and regulation through AU-rich elements. Interestingly, a recent study provided evidence for involvement of miRNAs in AU-rich element-mediated mRNA decay [29]).

**Table 4:**
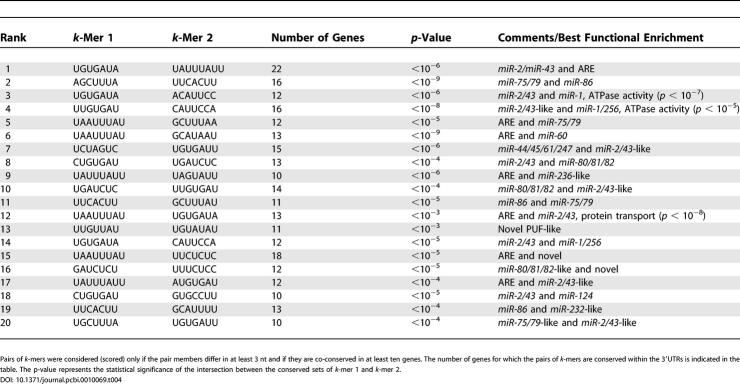
Top 20 Most Conserved *k*-Mer Co-occurrences in Worms

We also looked at whether coconserved sites are significantly clustered within their 3′UTRs. To do so, we calculated the median distance between the conserved occurrences within the 3′UTRs of the reference species (*C. elegans* for worms and *D. melanogaster* for flies). To calculate a statistical significance, we randomly selected the same number of pairs of positions within the same 3′UTRs, 10,000 times, and from this we calculated the null distribution of median distances. We found that most interactions were not associated with any statistically significant clustering. This may be due to the fact that pairs of *k*-mers are coconserved in relatively few genes, preventing strongly significant statistical assertions. We found that, in worms, the target sites for *miR-86* (UUCACUU) and *miR-87/miR-233/miR-356* (UUGCUCA) are significantly clustered (*p* < 10^−4^), with a median distance of 35 nt between coconserved occurrences in *C. elegans*. In another case, also in worms, the target sites for two coconserved and distinct miRNAs (or miRNA sets) *miR-2/miR-43* (CUGUGAU) and *miR-80/miR-81/miR-82* (UGAUCUC), were found to overlap more often than expected by chance (*p* < 10^−5^) (note that we limited the extent of the overlap to 4 nt in our coconservation analysis). This may be related to the as-yet unexplained observation that, in many cases, several distinct miRNAs appear to target the same site (see Tables 1 and S2).

### Discovery of Novel miRNAs

Starting from our list of highly conserved known and putative mRNA regulatory elements (*k*-mers), we systematically searched the *C. elegans* and *D. melanogaster* genomes for novel miRNAs that might target them. Our goal was to find candidate novel miRNAs that have not been found by previous approaches. We relaxed some assumptions about the structure of the miRNA precursor stem-loops and their pattern of conservation (e.g., see [42]), while introducing a new one, i.e., one having a 5′ extremity with perfect complementarity to at least one of our high-scoring *k*-mers. Briefly (see Materials and Methods for more details), we searched only for conserved stem-loop–forming sequences with a folding strength above a selected threshold, in which both orthologous stem-loops are required to yield a mature miRNA with a 5′ extremity complementary to the same high-scoring *k*-mer. We use this stringent condition when we refer to conservation of candidate miRNAs.

In worms, we obtained 80 candidate miRNAs that meet the (stringent) requirements described in Materials and Methods, with 30 of them being known *C. elegans* miRNAs. Note that the number of known miRNAs that are conserved (using only the conservation of the 80-nt stem-loop precursor sequence at the same threshold as described in Materials and Methods) between *C. elegans* and *C. briggsae* is 49; therefore, our miRNA discovery procedure for discovering highly conserved miRNAs has a 61% sensitivity in worms.

The top 30 worm miRNAs, ranked by decreasing folding strength (increasing ΔG), are listed in Table 5. They include 17 previously known miRNAs, the rest being candidate novel miRNAs. Table 5 shows that the candidate novel miRNAs are very similar to the known ones, in terms of ΔG, conservation in *C. briggsae,* and location within the genome (intergenic region or introns). Note that, although they were derived from *k*-mers lying in 3′UTRs of genes, none of the candidate novel miRNAs lie across from 3′UTRs. The 30 highest-scoring fly miRNAs, of which ten are known, are listed in Table S5. While all our candidate miRNAs should be systematically verified by experiments, our study suggests that the total number of miRNAs in metazoans may be much higher than previously thought, a prediction that agrees with a recent one made in mammalian genomes [13]. A conservative estimate based on the top 30 miRNAs discovered by our procedure would predict that the total number of miRNAs in worms is almost twice the current number in the miRNA registry. A less conservative estimate based on our 80 predicted miRNAs would indicate between 300 and 400 miRNAs in worms.

**Table 5:**
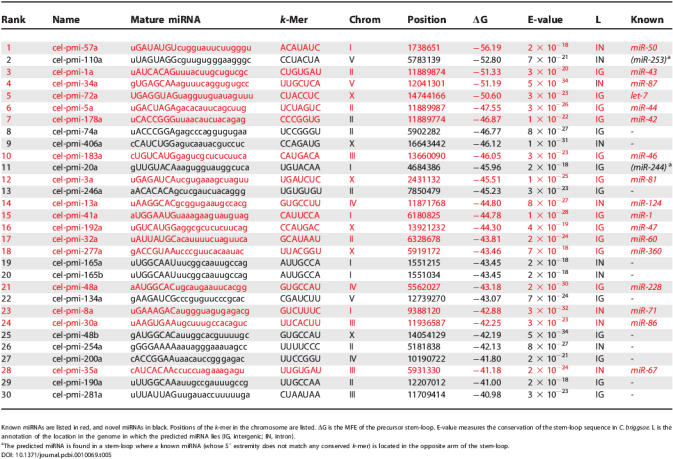
Top 30 Predicted *C. elegans* miRNAs, Sorted by ΔG

### Comparison of 3′UTR Regulation Between Worm and Fly

The intersection between the 442 and 497 ungapped highest-scoring worm and fly *k*-mers consists of 79 *k*-mers (19 of them complementary to the 5′ extremity of miRNAs in both organisms). This overlap is much higher than expected by chance (*p* < 10^−37^), indicating significant conservation of posttranscriptional regulatory sequences between these two phylogenetic groups. However, we found no significant overlap between the gene sets bearing the same *k*-mers in *C. elegans* and *D. melanogaster*. These results strongly resemble the ones we obtained for transcription factor binding sites [15], indicating that the regulators (miRNAs and RNA-binding proteins) are highly conserved, hence the sequences they bind are conserved; however, the sets of genes regulated by those regulators appear to differ significantly, indicating large-scale rewiring of the posttranscriptional regulatory network across large phylogenetic distances. This scenario is not surprising, since modifying the RNA-binding affinities of the regulators would cause drastic changes to the regulatory network topology, while appearance/deletion of single regulatory elements would presumably have much less drastic consequences for the cell. These results also indicate that motif discovery using the network-level conservation principle (at least as presented here) would fail if the compared species were very distantly related.

## Discussion

We have described an integrated approach for discovering conserved elements involved in posttranscriptional regulation and for predicting the miRNA regulators that may target these elements. Our approach is based on comparative genomics, but does not require the orthologous 3′UTRs to be aligned, and it requires only two genomes. Many of the regulatory elements we discovered were complementary to the 5′ extremity of known miRNAs, both in worms (in which we captured 62.4% of the known miRNAs) and in flies (62.0%). There may be several reasons why we failed to detect complementary *k*-mers for the remaining known miRNAs. It is possible that the sets of genes regulated by these miRNAs are small, decreasing the statistical power for detecting them. Alternatively, the corresponding posttranscriptional networks may have undergone extensive rewiring, rendering them undetectable by network-level conservation.

We have shown that the high-scoring *k*-mers are unlikely to be TFBSs (although we expect some level of contamination, due to transcription factors that bind downstream regions). Note, however, that some miRNAs may target mRNA sequences that match known transcription factor binding sites. For example, the target site of *C. elegans miR-248* matches the E-box, a site known to be bound by several transcription factors of the basic helix-loop-helix family; similarly, the target site of D. melanogaster miR-184* matches the binding site for GATA factors.

We have also shown that our approach conveniently defines sets of targets for miRNAs; a gene is predicted to be a target of a miRNA if the 3′UTR of the gene and its ortholog contain a globally conserved *k*-mer that is complementary to the 5′ extremity of the miRNA. While this simple approach appears to group together coherent sets of genes (as defined by functional enrichments and coexpression, for example), it has several limitations. For example, it does not predict *lin-41* as being a target of *let-7* in *C. elegans,* because this interaction involves a non-Watson-Crick pairing [43] and relatively extensive complementarity across the entire length of the miRNA/mRNA duplex. As recently shown in [8], inexact complementarity between the 5′ extremity of the miRNA and its target sequence can be rescued by a more extensive pairing across the length of the miRNA. Computational approaches for recovering these targets have been described elsewhere [10,44]. Also, our approach predicts only conserved targets; it is unclear what proportion of functional targets is not conserved between the species under consideration.

Since many of the most highly conserved *k*-mers do not match known miRNAs, we searched the *C. elegans* and *D. melanogaster* genomes for candidate novel miRNAs. We found many such putative miRNAs and showed that these predictions have all the features of known miRNAs. While experiments are now required to validate our predictions, a conservative estimate based on the highest-scoring *C. elegans* miRNAs indicates that there may be more than twice as many functional miRNAs in worms as is currently thought. A total of 101 (worms) and 110 (flies) of our high-scoring *k*-mers are complementary to the 5′ extremity of at least one known or novel miRNA. As illustrated in Table S1, we found that many of the remaining high-scoring *k*-mers extensively overlap with these sites (e.g., 120 and 147 of these remaining *k*-mers have at least 6-nt overlap with at least one *k*-mer that is complementary to the 5′ extremity of a known or novel miRNA). A small fraction (17 in worms, five in flies) of the remaining *k*-mers were complementary to miRNA 5′ extremities, if a single GU-pairing is allowed. Therefore, 204 *k*-mers in worms and 235 in flies are unaccounted for. As we have shown above, several of these *k*-mers are known to be protein-binding sites (e.g., by members of PUF family of RNA-binding proteins), and it is likely that many of the remaining *k*-mers are bound by RNA-binding proteins that have not been characterized yet. Finally, it is also possible that many of the remaining sites are targeted by miRNAs that are less conserved and/or those that interact weakly with their targets.

We have also shown that posttranscriptional regulatory networks have undergone extensive rewiring between worms and flies. The binding sites for miRNAs and known RNA-binding proteins are present in both phyla, suggesting that the regulators are still largely the same. However, these elements seem to be regulating entirely different sets of genes.

We envision several directions for further research. For example, our current approach does not make any assumptions about how miRNA or RNA-binding protein target sites evolve. We believe that, once the evolution of RNA regulatory elements is better understood, our approach may be refined to take RNA-specific modes of evolution into account, similarly to what was done with transcription factor binding sites [45]. Allowing more degeneracy within our RNA regulatory element representation also represents an interesting direction for further research, especially for RNA-binding protein target sites.

## Materials and Methods

The approach is outlined in Figure 1. It consisted of two main stages, the motif discovery stage (Figure 1A), in which FastCompare [15] was used to score exhaustive *k*-mer lists for network-level conservation, and the miRNA discovery stage (Figure 1B), in which novel miRNA candidates with 5′ complementarity to our high-scoring *k*-mers were predicted based on a combination of stem-loop structure and conservation.

### Data.

Entire genome sequences were downloaded from ENSEMBL [46]. The *D. pseudoobscura* genome sequence was obtained from [47]. We used real-length 3′UTRs, calculated according to ENSEMBL gene boundary coordinates for *C. elegans* and *D. melanogaster*. When several 3′UTRs were present for a single gene, only the longest one was retained. When no 3′UTR was present, we used 300 or 500 nt downstream of the stop codon, which correspond approximately to the 80th percentile of worm and fly 3′UTR lengths, respectively. For *C. briggsae* and *D. pseudoobscura* genes, we used 3′UTRs of the same length as those of their *C. elegans* and *D. melanogaster* orthologs, respectively. *C. elegans/C. briggsae* gene orthology relationships were obtained from Stein et al. [48]. *D. melanogaster/D. pseudoobscura* gene orthology relationships were mapped using reciprocal best BLAST hits. Functional annotations and GO classifications were downloaded from the GO web site (http://www.geneontology.org/). Functional enrichment *p*-values were calculated as described in [15,49]. Only functional enrichments with *p*-values lower than 10^−5^ are presented here.

### Motif finding using network-level conservation.

We applied FastCompare [15] to motif discovery using network-level conservation. FastCompare was modified for processing mRNA sequences (single-strand analysis). Briefly, each possible *k*-mer was considered as a candidate regulatory element. For each *k*-mer, we found the set of open reading frames in the first species that had at least one exact occurrence of the *k*-mer in their 3′UTR. We then found the set of open reading frames in the second species that had at least one occurrence of the same *k*-mer in their 3′UTR. The matches could be anywhere in the 3′UTRs: They do not have to be at the same positions in two orthologous 3′UTRs (as with multiple alignment). Since both functional and nonfunctional elements are expected to be conserved between two closely related species, the two sets are expected to overlap. However, under the network-level conservation principle, the extent of the overlap will be much greater for *k*-mers that represent functional mRNA regulatory elements. The strength of the overlap was measured using the hypergeometric distribution, which defines the probability of drawing two sets of size *s*_1_ and *s*_2_, having *i* or more elements in common, from a set of *N* elements, and this probability is given by:


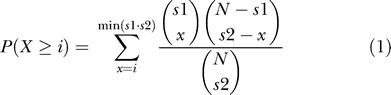


It is important to note that, due to basal conservation (that is, conservation arising from common ancestry), the hypergeometric *p*-values will generally be very small for most *k*-mers. Therefore, we use only these *p*-values as relative measures of network-level conservation and focus on *k*-mers with the greatest conservation. For simplicity, we define the “conservation score” as the negative logarithm (base *e*) of the hypergeometric *p*-value obtained for a given *k*-mer. Conservation scores were normalized for unequal lengths among 3′UTRs by weighing the contribution of each 3′UTR by 1/*length*, where *length* represents the length (in nt) of the 3′UTR. The variables *s*_1_, *s*_2_, and *i* were obtained by multiplying the corresponding weighted counts by 300 (for worms) and 500 (for flies), then rounding to the nearest integer. Further details about the motif discovery method are described in [15].

We calculated a conservation score for all possible *k*-mers, with *k* ranging from 7 to 9. We used randomized sequences to show that the high conservation scores obtained in this study were unlikely to be obtained by chance. To generate randomized pairs of orthologous 3′UTRs with the same level of divergence as in the actual data, we aligned each pair of orthologous 3′UTRs using ClustalW [50], and used the alignments to obtain an estimate of the substitution rates between the orthologous sequences. Starting from one of the orthologs, we created a randomized ortholog by mutating the initial sequence according to the estimated substitution frequencies. We then repeated FastCompare on the randomized sequences.

In the present study, we retained the 500 most conserved 7-mers (obtained from the actual sequences) and processed them as described in [15]. Briefly, we first extended 7-mers into 8-mers if, for a given 7-mer, there existed an 8-mer with a higher conservation score such that the 8-mer contained the 7-mer. We extended 8-mers into 9-mers in the same way. We also retained high-scoring 8-mers and 9-mers that did not have any substrings among 7-mers. Then we systematically removed *k*-mers that had higher scoring substrings. We define the conserved set of a given *k*-mer as the set of genes whose 3′UTRs contained at least one conserved occurrence of the *k*-mer.

To estimate the expected number of miRNAs complementary to a set of *m* high-scoring *k*-mers, we chose *m k*-mers at random, with the same numbers of 7-, 8-, and 9-mers as in the original set. We then evaluated the number of known miRNAs complementary to at least one of these randomly selected *k*-mers. We repeated the same procedure 100 times, and calculated the average number of complementary miRNAs.

### Defining miRNA targets.

We defined the target set of a given miRNA as the union of all conserved sets corresponding to the highly conserved *k*-mers complementary to its 5′ extremity (complementarity had to begin within 1 nt of the 5′ extremity). To estimate the number of targets expected by chance for each miRNA, we generated pairs of randomized orthologous sequences retaining the same level of divergence as the original pairs of sequences, as described above. Conserved sets for all *k*-mers complementary to miRNAs were then determined for these randomly generated sequences and subsequently used to create pseudo-target sets for each miRNA. The randomization procedure was repeated 100 times. Then, for each miRNA, the average size and standard deviation of the 100 corresponding pseudo-target sets were calculated.

### miRNA discovery.

For each conserved *k*-mer, we searched through the entire *C. elegans* or *D. melanogaster* genome for occurrences of the reverse-complementary *k*-mer on both strands of DNA. For each occurrence, we took two windows of length 80 nt (potentially corresponding to the two possible candidate miRNAs lying on the two arms of the 80-nt stem-loop sequence). We folded each window using RNAfold from the Vienna Package [51] to give the fold with minimal folding energy (MFE). If the fold formed a single stem-loop and the MFE was less than −30 kcal/mol at 25 °C, we retained the sequence as a potential miRNA precursor. We then tested for conservation of the potentially novel miRNA by searching for its homolog. We used BLAST (blastn) to search the second genome *(C. briggsae* or *D. pseudoobscura)* for regions homologous to the 80-nt stem-loop sequence, requiring the best matching sequence to have an E-value below a cutoff corresponding to conservation of 40% of the known miRNAs for that species (10^−17^ for worms and 10^−29^ for flies). We also required that the best matching sequence contains the exact conserved *k*-mer above, and folds into a single stem-loop with MFE less than −30 kcal/mol at 25 °C. We removed miRNA candidates located in exons or on the opposite strand of exons. Candidate mature miRNAs were defined as 23-nt sequences, such that the conserved reverse-complementary *k*-mer began at the second nucleotide from the 5′ extremity. A candidate mature miRNA matched a known mature miRNA if the positions of their 5′ ends were located within 2 nt on the genome (because miRNAs are generally 21–23 nt long). For the miRNA precursor stem-loops with candidate miRNAs from both arms of the stem-loop, we chose the one that matched the more conserved *k*-mer as the more likely candidate, except in the case where one matched a known miRNA. We ranked the candidate miRNAs by the MFE of their precursor stem-loop. To minimize false-positives, we present only candidate miRNAs with MFEs smaller than −34 kcal/mol at 25 °C as our final list of high-confidence predictions (80 for *C. elegans* and 92 for *D. melanogaster*).

We named our predicted miRNAs (pmi) by a number followed by a letter (e.g., *cel-pmi-74a*). The number corresponds to the rank of the conserved *k*-mer matched by our predicted miRNA. The letter corresponds to the ordinal value from all *pmi* matching that *k*-mer as ranked by MFE. When our *pmi* corresponds to a known miRNA, both names are shown.

### Data and Web site.

The sequences, programs, and detailed results described in this paper are available at http://tavazoielab.princeton.edu/mirnas/.

## Supporting Information

Figure S1 Distribution of Conservation Scores for the *D. melanogaster*/*D. pseudoobscura* Analysis, on Actual and Randomized 3′UTR Sequences Actual sequences are depicted in red and randomized sequences in black. Scores corresponding to some of the known miRNA target sites and RNA-binding protein sites are indicated by arrows. The top portions of both distributions are not shown, for the purpose of presentation.
(72 KB PDF)

Figure S2 High-Scoring *k*-Mers Are Complementary to the 5′ Ends of Many miRNAs Number of complementary fly miRNAs as a function of initial number of retained 7-mers (A), and proportion of fly 7-mers complementary to the 5′ extremity of at least one fly miRNA (B), as a function of the conservation rank (using a sliding window of size 50).
(66 KB PDF)

Figure S3 Distribution of Distances (in Nucleotides) from the First Nucleotide of the *k*-Mer to the 5′ Extremity of the miRNA, for All Pairs of High-Scoring *k*-Mers/Complementary miRNAs The distribution clearly shows that complementarity between high-scoring fly *k*-mers and miRNAs occurs primarily at the 5′ extremity of the miRNAs.
(66 KB PDF)

Figure S4 Estimated Number of Targets for *D. melanogaster* miRNAs That Are Complementary to One or More of Our High-Scoring *k*-Mers These numbers correspond to the number of genes with a 3′UTR containing at least one conserved *k*-mer complementary to the 5′ extremity of the corresponding miRNA (i.e., number of predicted targets), minus the expected number of targets by chance. Expected numbers were obtained by running the same analysis using 100 pairs of randomized fly genomes with the same level of divergence as the original ones, and averaging the obtained number of targets over the 100 runs. The error bars correspond to two standard deviations.
(80 KB PDF)

Table S1 Illustrating the Redundancy Among Some of the Highest-Scoring Worm *k*-Mers The top box shows *k*-mers (and their ranks) that have a 1-nt difference with four of the highest-scoring *k*-mers. The bottom box shows *k*-mers (and their ranks) that have a 6-nt overlap with the same four *k*-mers.
(69 KB PDF)

Table S2 Fly *k*-Mers Complementary to 5′ Extremity of Known Fly miRNAs *k*-Mers are grouped by sequence similarity and overlap. Each *k*-mer within a group is complementary to (i.e., matches) at least one miRNA, indicated by a number. If the *k*-mer is also found within the list of highest conserved worm *k*-mers, its rank is given, and * indicates that the *k*-mer is also complementary to the 5′ extremity of a worm miRNA
(79 KB PDF)

Table S3 *k*-Mers Complementary to Known miRNAs, But Not Within the 5′ Extremity in Worms and Flies Complementary *k*-mers in worm (A) and fly (B) are listed.
(81 KB PDF)

Table S4 Top 20 Most Conserved *k*-Mer Co-occurrences in Flies Pairs of *k*-mers were considered (scored) only if the pair members differed in at least 3 nt and if they are coconserved in at least ten genes. The number of genes for which the pairs of *k*-mers were conserved within the 3′UTRs is indicated in the table. The *p*-value represents the statistical significance of the intersection between the conserved sets of *k*-mer 1 and *k*-mer 2.
(79 KB PDF)

Table S5 Top 30 Predicted *D. melanogaster* miRNAs, Sorted by ΔG Red miRNAs are known, black are novel. Pos is the position of the *k*-mer in the chromosome. ΔG is the MFE of the precursor stem-loop. E-value measures the conservation of the stem-loop sequence in *D. pseudoobscura*. L is the annotation of the location in the genome in which the predicted miRNA lies (IG, intergenic; IN, intron).
(82 KB PDF)

## Abbreviations

ARE: AU-rich element
GO: Gene Ontology
MFE: minimal folding energy
miRNA: microRNA
nt: nucleotide
TFBS: transcription factor binding site
UTR: untranslated region
