# Supplementary material for: Revealing Posttranscriptional Regulatory Elements Through Network-Level Conservation
Source: PLoS Comput Biol. 2005 Dec 9;1(7):e69. doi: 10.1371/journal.pcbi.0010069 (PMC1309705; doi:10.1371/journal.pcbi.0010069)

Figure S1. Distribution of conservation scores for the *D. melanogaster*/*D. pseudoobscura* analysis, on actual (red) and randomized (black) 3' UTR sequences. Scores corresponding to some of the known miRNA target sites and RNA-binding protein sites are indicated by arrows. The top portions of both distributions are not shown, for the purpose of presentation.

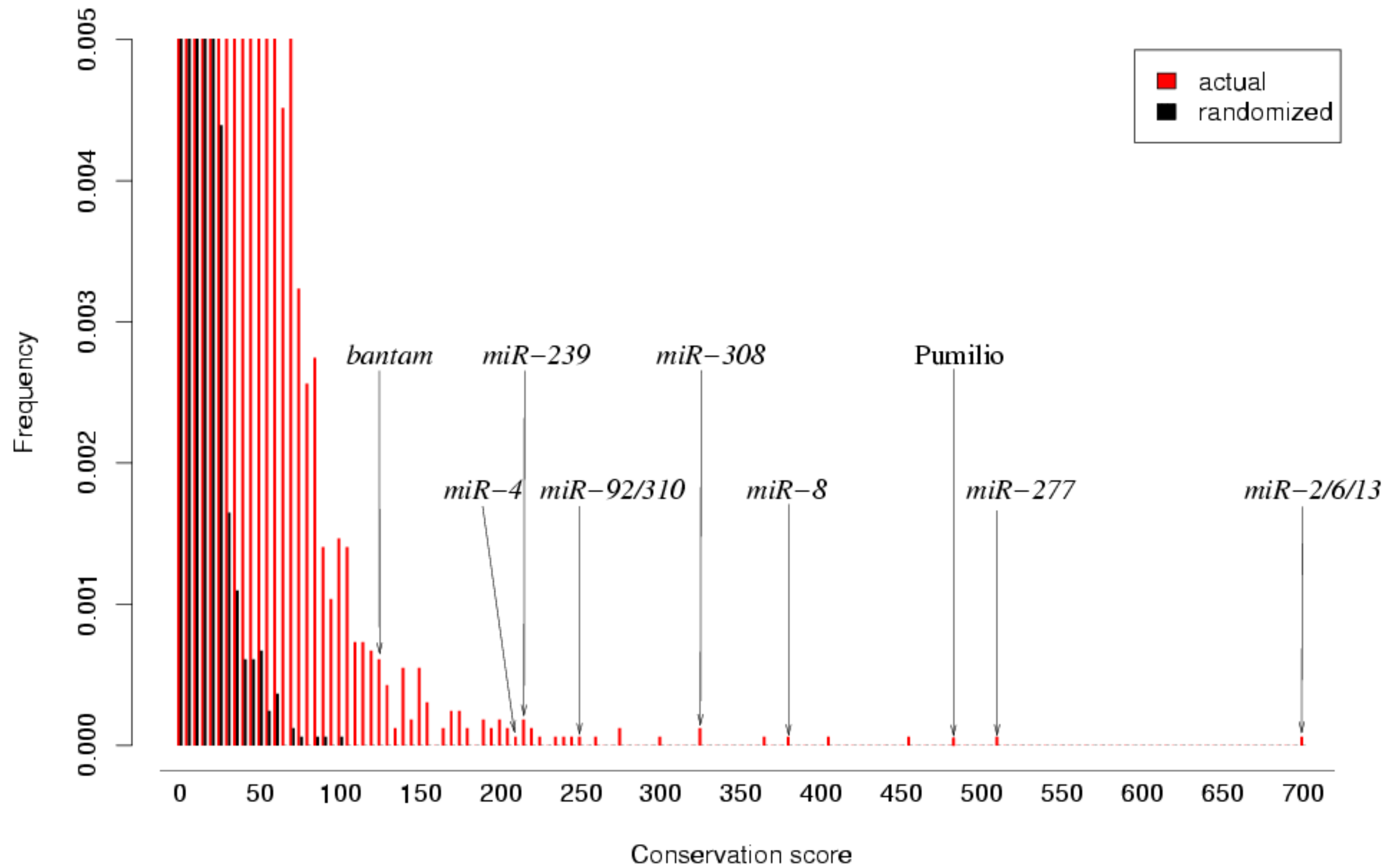

Supplement: Supplementary file 1 [file pcbi.0010069.sg001.pdf]
