# Supplementary material for: Revealing Posttranscriptional Regulatory Elements Through Network-Level Conservation
Source: PLoS Comput Biol. 2005 Dec 9;1(7):e69. doi: 10.1371/journal.pcbi.0010069 (PMC1309705; doi:10.1371/journal.pcbi.0010069)

Figure S2. (A) number of complementary fly miRNAs as a function of initial number of retained 7-mers, (B) proportion of fly 7-mers complementary to the 5' extremity of at least one fly miRNA, as a function of the conservation rank (using a sliding window of size 50).

(A)

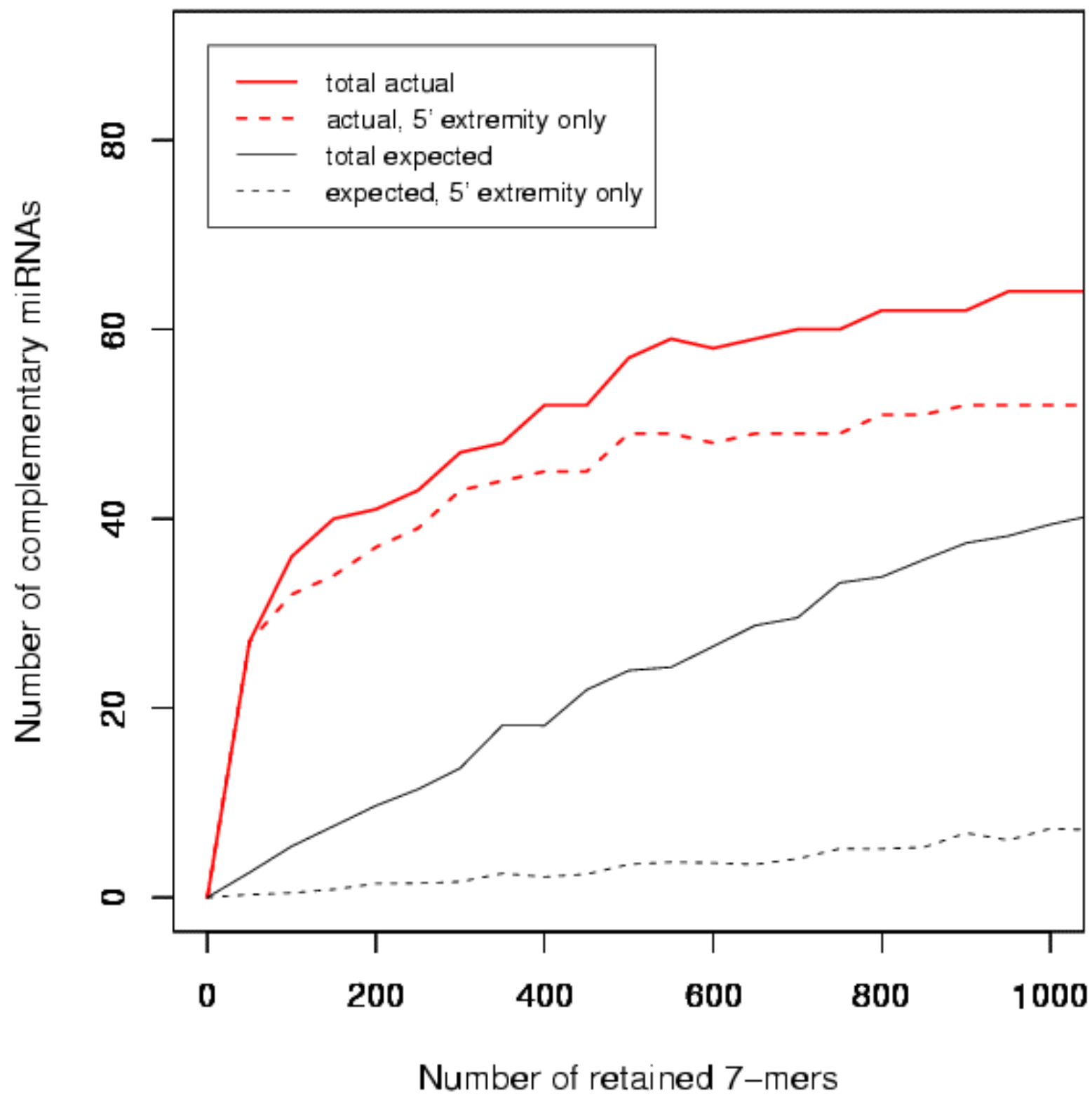

(B)

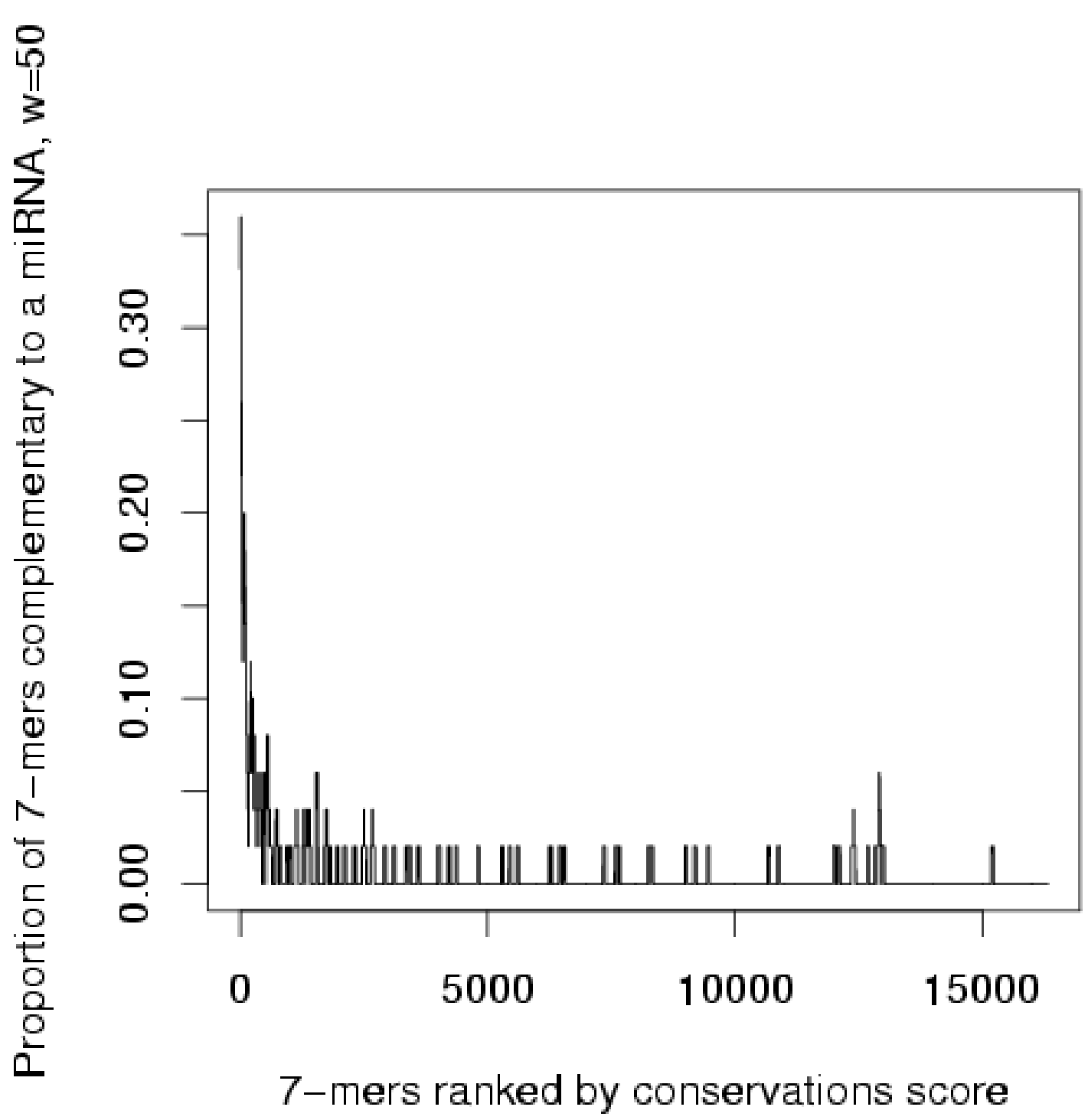

Supplement: Supplementary file 2 [file pcbi.0010069.sg002.pdf]
