# Supplementary material for: Revealing Posttranscriptional Regulatory Elements Through Network-Level Conservation
Source: PLoS Comput Biol. 2005 Dec 9;1(7):e69. doi: 10.1371/journal.pcbi.0010069 (PMC1309705; doi:10.1371/journal.pcbi.0010069)

Figure S3. Distribution of distances (in nucleotides) from the first nucleotide of the *k*-mer to the 5' extremity of the miRNA, for all pairs of high scoring *k*-mers / complementary miRNAs. The distribution clearly shows that complementarity between high scoring fly *k*-mers and miRNAs occurs primarily at the 5' extremity of the miRNAs.

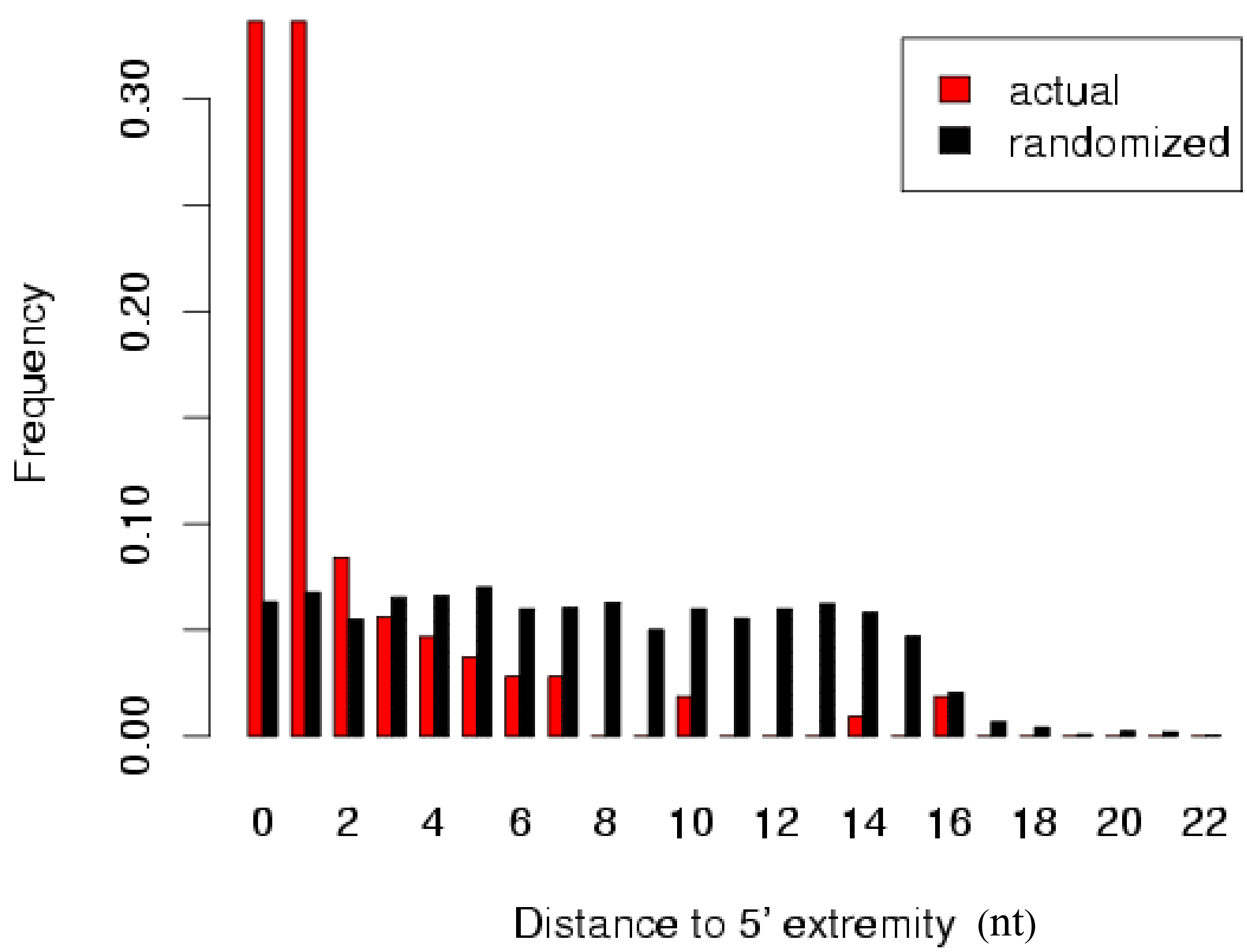

Supplement: Supplementary file 3 [file pcbi.0010069.sg003.pdf]
