# Supplementary material for: Revealing Posttranscriptional Regulatory Elements Through Network-Level Conservation
Source: PLoS Comput Biol. 2005 Dec 9;1(7):e69. doi: 10.1371/journal.pcbi.0010069 (PMC1309705; doi:10.1371/journal.pcbi.0010069)

Figure S4. Estimated number of targets for *D. melanogaster* miRNAs that are complementary to at least one of our high scoring *k*-mers; these numbers correspond to the number of genes whose 3'UTR contains at least one conserved *k*-mer complementary to the 5' extremity of the corresponding miRNA (*i.e.* number of predicted targets), minus the expected number of targets by chance. Expected numbers were obtained by running the same analysis using 100 pairs of randomized fly genomes with the same level of divergence as the original ones, and averaging the obtained number of targets over the 100 runs. The error bars correspond to two standard deviations.

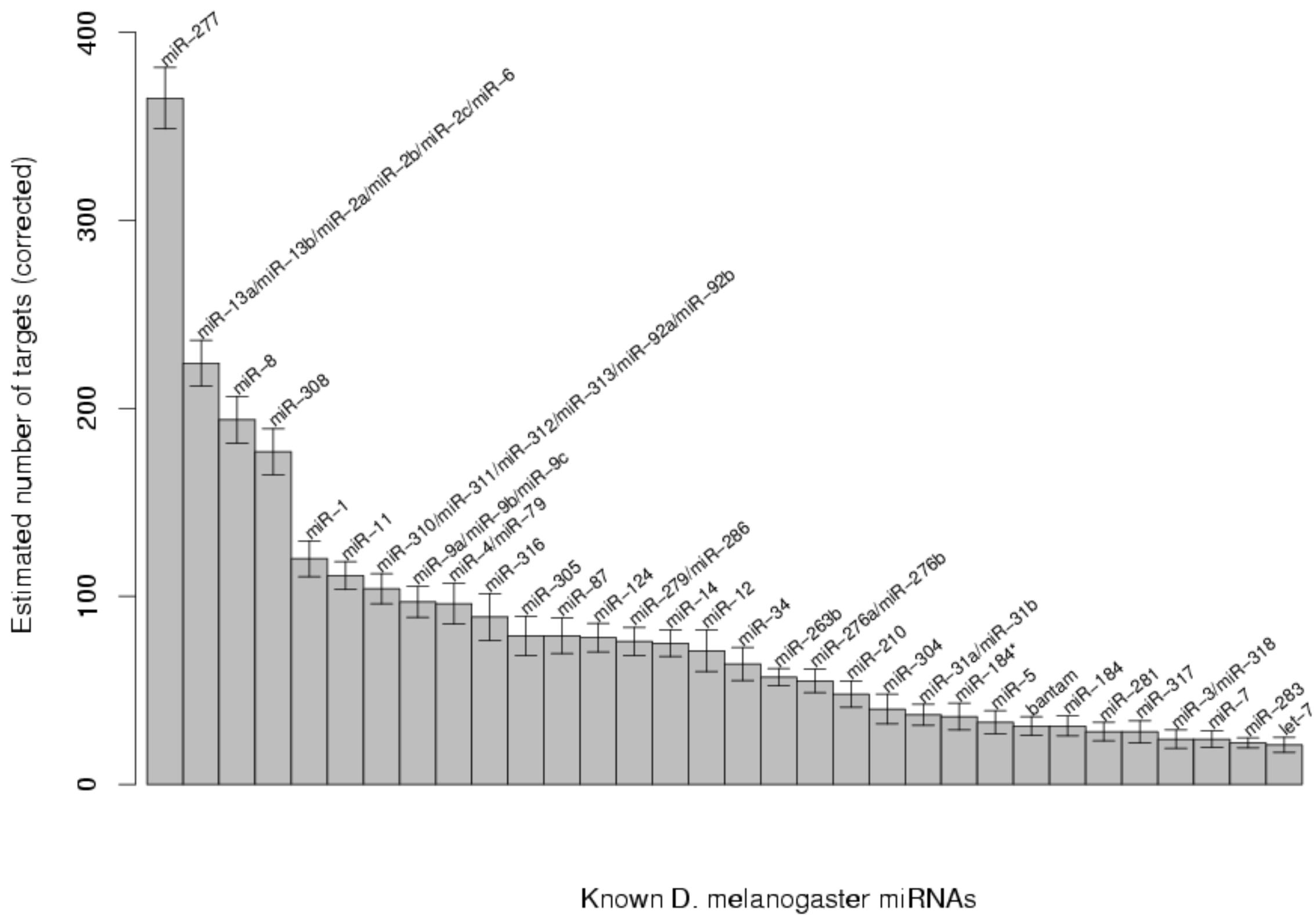

Supplement: Supplementary file 4 [file pcbi.0010069.sg004.pdf]
