# Supplementary material for: Revealing Posttranscriptional Regulatory Elements Through Network-Level Conservation
Source: PLoS Comput Biol. 2005 Dec 9;1(7):e69. doi: 10.1371/journal.pcbi.0010069 (PMC1309705; doi:10.1371/journal.pcbi.0010069)
Supplement: Supplementary file 5 [file pcbi.0010069.st001.pdf]

Table S1. Illustrating the redundancy among some of the highest scoring worm *k*-mers. The top box shows *k*-mers (and their ranks) that have 1 nt difference with four of the highest scoring *k*-mers. The bottom box shows *k*-mers (and their ranks) that have a 6 nt overlap with the same four *k*-mers.

**CUGUGAU**	1	**UGAUCUC**	**3**	**UGUAAAUA**	**9**	**ACAUUCC**	**12**
U......	35	C......	58	.....U..	130	U......	47
A......	67	A......	61	......A.	300	C......	159
.....U.	164	......U	81			G......	220
G......	166	G......	121				
......A	322	.C.....	374				
....U..	423	......A	405				

**CUGUGAU**	1	**UGAUCUC**	**3**	**UGUAAAUA**	**9**	**ACAUUCC**	**12**
-......A	7	-......A	14	C.......-	223	-......A	41
-......U	49	-......U	17			-......C	99
-......C	109	A......-	91			-......U	103
-......G	345	-......G	129			-......G	256
		-......C	412				
