# Supplementary material for: Revealing Posttranscriptional Regulatory Elements Through Network-Level Conservation
Source: PLoS Comput Biol. 2005 Dec 9;1(7):e69. doi: 10.1371/journal.pcbi.0010069 (PMC1309705; doi:10.1371/journal.pcbi.0010069)
Supplement: Supplementary file 6 [file pcbi.0010069.st002.pdf]

Table S2. Fly *k*-mers complementary to 5' extremity of known fly miRNAs. *k*-mers are grouped by sequence similarity and overlap. Each *k*-mer within a group is complementary to (*i.e.* matches) at least one miRNA, indicated by a number. If the *k*-mer is also found within the list of highest conserved worm *k*-mers, its rank is given, and * indicates that the *k*-mer is also complementary to the 5' extremity of a worm miRNA.

Rank	Worm rank	*k*-mers	Matches	complementary miRNAS		
1	7*	UGUGAUA	1-6	(1)	*miR-2a*	**UAUCACAg**ccagcuuugaugagc
9	49	UGUGAUU	7	(2)	*miR-2b*	**UAUCACAg**ccagcuuugaggagc
10	1*	CUGUGAU	1-7	(3)	*miR-2c*	**UAUCACAg**ccagcuuugaugggc
				(4)	*miR-6*	**UAUCACAg**uggcuguucuuuuu
				(5)	*miR-13a*	**UAUCACAg**ccauuuugaugagu
				(6)	*miR-13b*	**UAUCACAg**ccauuuugaugagu
				(7)	*miR-308*	**AAUCACAg**gauuauacugugag
3	-	GCAUUUA	1	(1)	*miR-277*	**UAAAUGCa**cuaucugguacgaca
6	107*	UGCAUUU				
7	-	AGUAUUA	1	(1)	*miR-8*	**UAAUACUg**ucagguaaagauguc
37	132*	CAGUAUU				
13	23*	CUAGUCA	1,2	(1)	*miR-279*	**UGACUAGa**uccacacucauuaa
27	5*	UCUAGUC		(2)	*miR-286*	**UGACUAGa**ccgaacacucgugcu
14	19*	GUGCAAU	1-6	(1)	*miR-92a*	c**AUUGCAC**uugucccggccuau
				(2)	*miR-92b*	a**AUUGCAC**uagucccggccugc
				(3)	*miR-310*	u**AUUGCAC**acuucccggccuuu
				(4)	*miR-311*	u**AUUGCAC**auucaccggccuga
				(5)	*miR-312*	u**AUUGCAC**uugagacggccuga
				(6)	*miR-313*	u**AUUGCAC**uuuucacagcccga
15	12*	ACAUUCC	1	(1)	*miR-1*	u**GGAAUGU**aaagaaguauggag
42	41*	CAUUCCA				
23	27*	AGCUUUA	1,2	(1)	*miR-4*	a**UAAAGCU**agacaaccauuga
				(2)	*miR-79*	**UAAAGCU**agauuaccaaagcau
28	13*	GUGCCUU	1	(1)	*miR-124*	u**AAGGCAC**gcggugaaugccaag
116	-	UGCCUUA				
29	-	CCAAAGA	1-3	(1)	*miR-9a*	**UGUUUGG**uuaucuagcuguauga
				(2)	*miR-9b*	**UGUUUGG**ugauuuuagcuguaug
				(3)	*miR-9c*	**UGUUUGG**uauucuagcuguaga
31	160	GUGCCAA	1	(1)	*miR-263b*	c**UUGGCAC**ugggagaauucac
38	-	AGACUGA	1	(1)	*miR-14*	**UCAGUCUu**uuucucucuccua
51	-	AAGACUG				
52	-	AAAAGAC	1	(1)	*miR-316*	u**GUCUUUU**uccgcuuacuggcg
77	-	AAAGACA				
64	-	AGUACAA	1	(1)	*miR-305*	a**UUGUACU**ucaucaggugcucug
435	-	GUACAAU				
89	14*	GAUCUCA	1	(1)	*bantam*	**UGAGAUC**auuuugaaagcugauu
93	-	CCGUCCA	1	(1)	*miR-184*	**UGGACGGa**gaacugauaagggc
283	-	UCCGUCC				
98	34*	UUGCUCA	1	(1)	*miR-87*	u**UGAGCAA**aauuucaggugug
118	93*	UGCUCAA				
105	-	UGAUAUUUA	1	(1)	*miR-283*	**UAAAUAUCA**gcugguaauucu
110	441	ACUGCCA	1	(1)	*miR-34*	**UGGCAGUg**ugguuagcugguug
218	202*	CACUGCC				
126	345*	UGUGAUG	1	(1)	*miR-11*	**CAUCACA**gucugaguucuugc
143	-	GUUCCUA	1,2	(1)	*miR-276a*	**UAGGAACu**ucauaccgugcucu
369	-	AGUUCCU		(2)	*miR-276b*	**UAGGAACu**uaauaccgugcucu
188	-	GUCUUCC	1	(1)	*miR-7*	u**GGAAGAC**uagugauuuuguugu
196	-	GUGUUCA	1	(1)	*miR-317*	**UGAACAC**agcuggugguauccagu
201	-	AUACUCA	1	(1)	*miR-12*	**UGAGUAUu**acaucagguacuggu
321	-	AAUACUC				
238	-	UGAGAUU	1	(1)	*miR-304*	u**AAUCUCA**auuuguaaaugugag
241	66*	UCUUGCC	1,2	(1)	*miR-31a*	u**GGCAAGA**ugucggcauagcuga
				(2)	*miR-31b*	u**GGCAAGA**ugucggaauagcug
289	183*	CAUGACA	1	(1)	*miR-281*	**UGUCAUG**gaauugcucucuuugu
314	211*	UACCUCA	1	(1)	*let-7*	**UGAGGUA**guagguuguauagu
387	-	GUUCCUU	1	(1)	*miR-5*	a**AAGGAAC**gaucguugugauaug
488	-	ACGCACA	1	(1)	*miR-210*	u**UGUGCGU**gugacagcggcua
490	-	CCAGUGA	1,2	(1)	*miR-3*	**UCACUGG**gcaaagugugucuca
				(2)	*miR-318*	**UCACUGG**gcuuuguuuaucuca
497	-	UGAUAAG	1	(1)	*miR-184**	c**CUUAUCA**uucucucgccccg
