# Supplementary material for: Revealing Posttranscriptional Regulatory Elements Through Network-Level Conservation
Source: PLoS Comput Biol. 2005 Dec 9;1(7):e69. doi: 10.1371/journal.pcbi.0010069 (PMC1309705; doi:10.1371/journal.pcbi.0010069)
Supplement: Supplementary file 7 [file pcbi.0010069.st003.pdf]

Table S3. *k*-mers complementary to known miRNAs, but not within the 5' extremity in worms (A) and flies (B).

(A)

Rank	Fly rank	*k*-mers	complementary miRNAS	
20	-	UGUACAA	*miR-244*	ucuuugg**uuguacaaag**ugguaug
38	40	UUGUACA		
89	-	UUUGUAC		
439	368	CUUUGUA		
63	-	UUACGGG	*miR-54*	ua**cccguaa**ucuucauaauccgag
			miR-56	ua**cccguaa**uguuuccgcugag
78	-	AUGUACA	*miR-40*	ucaccgg**guguacau**cagcuaa
108	-	UGUACAC		
80	-	GGUCUCA	*lin-4*	uccc**ugagacc**ucaaguguga
316	-	AGGUCUC		
110	-	CCUACUA	*let-7*	ugagg**uaguagg**uuguauaguu
149	148	UGCCAUU	*miR-74*	uggcaaga**aauggcagu**cuaca
441	110*	ACUGCCA		
150	-	UGUGCAU	*miR-60*	uauu**augcaca**uuuucuaguuca
190	121	UUGCCAA	*miR-266*	aggcaagacu**uuggcaaa**gc
241	277	UUUGCCA	*miR-269*	ggcaagacuc**uggcaaa**acu
199	-	UGUAGAU	*miR-42*	caccggguuaac**aucuaca**g
91	-	AUGAUCU	*miR-80*	ug**agaucau**uagu**ugaaagc**cga
204	-	GCUUUCA	*miR-81*	ug**agaucau**cg**ugaaagc**uagu
360	-	AGCUUUC	*miR-82*	ug**agaucau**cg**ugaaagc**cagu
221	-	CCAUGUC	*miR-71*	ugaaa**gacaugg**guaguga
247	-	UCUCAGG	*miR-237*	uc**ccugaga**auucucgaacagcuu
268	-	AACUGCC	*miR-73*	uggcaagaugua**ggcaguu**cagu
277	-	UUACGGU	*miR-360*	ug**accguaa**ucccguucacaa
280	-	ACGGUUC	*miR-61*	ugacua**gaaccgu**uacucaucuc
318	169*	AGUGCCA	*miR-228*	aa**uggcacu**gcaugaauucacgg
362	-	UUUCUAG	*miR-67*	ucacaacccuc**cuagaaa**gaguaga
379	-	CCAGUGU	*miR-229*	aaug**acacugg**uuaucuuuuccaucgu
404	-	UAUGUGUA	*miR-239a*	uuuguac**uacacaua**gguacugg
429	-	UUUCUCC	*miR-38*	ucaccg**ggagaaaaa**cuggagu
433	-	AGGUUGU	*miR-67*	uc**acaaccu**ccuagaaagaguaga
436	-	CAUCAUC	*miR-59*	ucgaaucguuuaucag**gaugaug**

(B)

rank	Worm rank	*k*-mers	complementary miRNAS	
55	-	AAAGACU	*miR-14*	uc**agucuuu**uucucucuccua
65	-	GUAUUAA	*miR-276b*	uaggaac**uuaauac**cgugcucu
73	-	GUGCAUU	*miR-277*	ua**aaugcac**uaucugguacgaca
77		AAAAGAC	*miR-14*	uca**gucuuuu**ucucucuccua
82	-	GUGCAAA	*miR-287*	uguguugaaaaucg**uuugcac**
83	-	AACCAAA	*miR-9a*	ug**uuugguu**aucuagcuguauga
85	136*	CAUAUCA	*miR-5*	aaaggaacgaucguug**ugauaug**
91	-	UACCAAA	*miR-9c*	ug**uuuggua**uucuagcuguaga
95	48*	GUGCCAU	*miR-263a*	guu**aauggcacu**ggaagaauucac
148	149*	UGCCAUU		
169	318*	AGUGCCA		
133	-	AGCAACA	*miR-315*	uuuuga**uuguugcu**cagaaagc
334	-	GCAACAA		
134	-	ACAACAA	*miR-7*	uggaa**gacuagu**gauu**uguugu**
391	334	ACUAGUC		
141		AAGUGCA	*miR-92a*	cau**ugcacuug**ucccggccuau
300	-	CAAGUGC	*miR-312*	uau**ugcacuug**agacggccuga
			miR-313	uau**ugcacuu**uucacagcccga
169	318*	AGUGCCA	*miR-263b*	cu**uggcacu**gggagaauucac
254	-	UGUCUAG	*miR-4*	auaaag**cuagaca**accauuga
444	-	UUGUCUA		
338	-	CAAAUUG	*miR-304*	uaaucu**caauuug**uaaaugugag
361	-	AUUCCAU	*miR-281*	uguc**auggaau**ugcucucuuugu
372	-	AGAGGCU	*miR-282*	aaucu**agccucu**acuaggcuuugucugu
375		CAAUGCA	*miR-33*	agg**ugcauug**uagucgcauug
381	-	CUAAGUA	*miR-306*	ucagg**uacuuag**ugacucucaa
464	-	AGUCACU		
389	223	UAUGCAA	*miR-280*	uguauuuacg**uugcaua**ugaaaugaua

rank	Worm rank	*k*-mers	complementary miRNAS	
55	-	AAAGACU	*miR-14*	uc**agucuuu**uucucucuccua
65	-	GUAUUAA	*miR-276b*	uaggaac**uuaauac**cgugcucu
73	-	GUGCAUU	*miR-277*	ua**aaugcac**uaucugguacgaca
77		AAAAGAC	*miR-14*	uca**gucuuuu**ucucucuccua
82	-	GUGCAAA	*miR-287*	uguguugaaaaucg**uuugcac**
83	-	AACCAAA	*miR-9a*	ug**uuugguu**aucuagcuguauga
85	136*	CAUAUCA	*miR-5*	aaaggaacgaucguug**ugauaug**
91	-	UACCAAA	*miR-9c*	ug**uuuggua**uucuagcuguaga
95<br>148<br>169	48*<br>149*<br>318*	GUGCCAU<br>UGCCAUU<br>AGUGCCA	*miR-263a*	guu**aauggcacu**ggaagaauucac
133<br>334	-<br>-	AGCAACA<br>GCAACAA	*miR-315*	uuuuga**uuguugcu**cagaaagc
475	-	UUGGACA	*miR-219*	ugau**uguccaa**acgcaauucuug
