# Supplementary material for: Revealing Posttranscriptional Regulatory Elements Through Network-Level Conservation
Source: PLoS Comput Biol. 2005 Dec 9;1(7):e69. doi: 10.1371/journal.pcbi.0010069 (PMC1309705; doi:10.1371/journal.pcbi.0010069)
Supplement: Supplementary file 8 [file pcbi.0010069.st004.pdf]

Table S4. Top 20 most conserved *k*-mer co-occurrences in flies. Pairs of *k*-mers were considered (scored) only if the pair members differ in at least 3 nt and if they are co-conserved in at least 10 genes. The number of genes for which the pairs of *k*-mers are conserved within the 3'UTRs is indicated in the table. The p-value represents the statistical significance of the intersection between the conserved sets of *k*-mer 1 and *k*-mer 2.

Rank	*k*-mer 1	*k*-mer 2	#genes	p-value	Comments / Best functional enrichments
1	UGUGAUA	UAUUUAUU	29	$<10^{-10}$	*miR-2a/2b/2c/6/13a/13b* site and ARE
2	UGUGAUA	AGCUUUA	24	$<10^{-17}$	*miR-2a/2b/2c/6/13a/13b* and *miR-277*, transcriptional repressor activity ($p<10^{-8}$)
3	UAUUUAUU	AGUAUUA	38	$<10^{-19}$	ARE and *miR-8*
4	CACACAC	UAUAUAUA	120	$<10^{-127}$	Novel sites, organ development ($p<10^{-9}$)
5	UAUUUAUU	UGUAAAUA	44	$<10^{-19}$	ARE and PUF
6	UAUUUAUU	UGCAUUU	43	$<10^{-15}$	ARE and *miR-277*
7	CACACAC	UAUAUAC	106	$<10^{-97}$	Novel sites, organ development ($p<10^{-9}$)
8	UAUUUAUU	GUGAUAU	19	$<10^{-8}$	ARE and novel
9	UGUGAUA	UAUUUAUA	21	$<10^{-10}$	*miR-2a/2b/2c/6/13a/13b* site and ARE
10	UGCAUUU	UUGUUAA	35	$<10^{-19}$	*miR-277* and novel
11	CAGCAGC	ACAACAA	63	$<10^{-55}$	Novel sites, regulation of transcription($p<10^{-9}$)
12	UGCAUUU	UUGUUAU	32	$<10^{-14}$	*miR-277* and novel
13	UGCAUUU	UAAUUUAU	32	$<10^{-15}$	*miR-277* and ARE
14	UGUAAAUA	UAAUUGUA	18	$<10^{-13}$	PUF and novel
15	AGUAUUA	GUGAUAU	19	$<10^{-12}$	*miR-8* and novel
16	GCAUUUA	UUAGCAU	23	$<10^{-23}$	*miR-277* and novel
17	UGCAUUU	CACACAC	65	$<10^{-40}$	*miR-277* and novel, organ development ($p<10^{-9}$)
18	UAUAUAC	AACCAAA	85	$<10^{-71}$	Novel and *miR-9a-like*
19	AGUAUUA	UUUAGUU	38	$<10^{-21}$	*miR-8* and novel, neurogenesis ($p<10^{-7}$)
20	CACACAC	UUUAGUU	79	$<10^{-58}$	*N*ovel sites, organ development ($p<10^{-9}$)
