# Supplementary material for: Revealing Posttranscriptional Regulatory Elements Through Network-Level Conservation
Source: PLoS Comput Biol. 2005 Dec 9;1(7):e69. doi: 10.1371/journal.pcbi.0010069 (PMC1309705; doi:10.1371/journal.pcbi.0010069)
Supplement: Supplementary file 9 [file pcbi.0010069.st005.pdf]

Table S5. Top 30 predicted *D. melanogaster* miRNAs, sorted by ΔG. Red miRNAs are known, black ones are novel. Pos is the position of the *k*-mer in the chromosome. ΔG is the MFE of the precursor stem-loop. E-value measures the conservation of the stem-loop sequence in *D. pseudoobscura*. L is the annotation of the location in the genome in which the predicted miRNA lies (IN is intron; IG is intergenic).

Rank	Name	Mature miRNA	k-mer	Chrom	Pos	ΔG	E-value	L	Known
1	dme-pmi-1a	aUAUCACAgccagcuuugaugag	UGUGAUA	2L	19565440	-47.20	7E-37	IN	*miR-2a-2*
2	dme-pmi-1b	aUAUCACAgccagcuuugaggag	UGUGAUA	2L	19566133	-45.76	7E-37	IN	*miR-2b-2*
3	dme-pmi-408a	gAUUGCCAuugccguugcuguug	UGGCAAU	3R	12511982	-45.41	2E-31	IN	-
4	dme-pmi-79a	uGCAACAAuuugcagcaaauuga	UUGUUGC	3R	22996489	-44.82	4E-29	IG	-
5	dme-pmi-201a	uUGAGUAUuacaucagguacugg	AUACUCA	X	15343415	-44.16	2E-31	IN	*miR-12*
6	dme-pmi-116a	cUAAGGCAauuuaugauugaccg	UGCCUUA	X	6283186	-44.10	3E-36	IG	-
7	dme-pmi-3a	gUAAAUGCacuaucugguacgac	GCAUUUA	3R	5925801	-44.04	4E-29	IG	*miR-277*
8	dme-pmi-488a	uUGUGCGUgugacagcggcuauu	ACGCACA	X	17962208	-43.87	2E-34	IG	*miR-210*
9	dme-pmi-24a	cUAAGUACuagugccgcaggagu	GUACUUA	3R	9290010	-43.06	4E-38	IN	-
10	dme-pmi-126a	cCAUCACAaccuccuugagugag	UGUGAUG	2R	5133091	-42.42	1E-38	IN	*miR-307*
11	dme-pmi-346a	gCAGCUGCagcagcgacagcggu	GCAGCUG	2R	12869266	-41.84	7E-34	IG	-
12	dme-pmi-140a	aAAAUGGCgcacauuaaaagcgg	GCCAUUU	3R	26758835	-41.36	1E-29	IG	-
13	dme-pmi-143a	gUAGGAACuucauaccgugcucu	GUUCCUA	3L	10339227	-40.97	3E-33	IG	*miR-276a*
14	dme-pmi-28a	uAAGGCACgcggugaaugccaag	GUGCCUU	2L	17562355	-40.82	1E-32	IG	*miR-124*
15	dme-pmi-151a	uUAUUGUCauuuauuguuguugu	GACAAUA	3L	15241046	-40.68	1E-29	IG	-
16	dme-pmi-23a	aUAAAGCUagauuaccaaagcau	AGCUUUA	2L	16694541	-40.13	1E-29	IN	*miR-79*
17	dme-pmi-3b	uUAAAUGCaccguuaacuacuuc	GCAUUUA	X	11590220	-40.12	2E-31	IN	-
18	dme-pmi-3c	uUAAAUGCaccguuaacuacuuc	GCAUUUA	X	11576952	-40.12	2E-31	IG	-
19	dme-pmi-71a	uGCUGCUGuuguugucgggccgu	CAGCAGC	3L	10996048	-39.97	3E-33	IG	-
20	dme-pmi-29a	gUCUUUGGuuaucuagcuguaug	CCAAAGA	3L	19530398	-39.71	1E-38	IG	*miR-9a*
21	dme-pmi-1c	uUAUCACAuucacaugcaaauug	UGUGAUA	2L	17153435	-38.86	4E-29	IG	-
22	dme-pmi-277a	cUGGCAAAcuccguuugacugcu	UUUGCCA	2L	17884567	-38.58	4E-29	IG	-
23	dme-pmi-365a	gCCAACAAauuaacugcgacaug	UUGUUGG	3R	27091354	-38.56	2E-31	IG	-
24	dme-pmi-74a	cAAUGCAUucgaucgucucucug	AAUGCAUU	3L	21723559	-38.44	4E-29	IG	-
25	dme-pmi-32a	gGUGUGUGugcucuaucagcgcc	CACACAC	3R	24403146	-38.06	1E-29	IG	-
26	dme-pmi-95a	uAUGGCACguuaaaguguuaaug	GUGCCAU	2R	15418600	-37.95	7E-34	IG	-
27	dme-pmi-14a	aAUUGCACauuucacguuguugc	GUGCAAU	X	20126099	-37.86	2E-31	IG	-
28	dme-pmi-71b	aGCUGCUGcuuuaaauuuaugcc	CAGCAGC	3L	5100430	-37.85	2E-31	IG	-
29	dme-pmi-59a	cUUGCACUuaauugccaauggcu	AGUGCAA	3R	25354567	-37.82	3E-33	IG	-
30	dme-pmi-145a	gAAAUUGCuggcaauucgcagag	GCAAUUU	2L	3576510	-37.62	6E-31	IG	-
